# Evolutionary relationships of the old world fruit bats (Chiroptera, Pteropodidae): Another star phylogeny?

**DOI:** 10.1186/1471-2148-11-281

**Published:** 2011-09-30

**Authors:** Francisca C Almeida, Norberto P Giannini, Rob DeSalle, Nancy B Simmons

**Affiliations:** 1 American Museum of Natural History, Division of Vertebrate Zoology, Department of Mammalogy, Central Park West at 79th Street, New York, NY 10024, USA; 2 American Museum of Natural History, Sackler Institute for Comparative Genomics, Central Park West at 79th Street, New York, NY 10024, USA; 3 Universitat de Barcelona, Departament de Genètica, Diagonal 645, Barcelona, 08028, Spain; 4 CONICET, Programa de Investigaciones de Biodiversidad Argentina, Universidad Nacional de Tucumán, Facultad de Ciencias Naturales e Instituto Miguel Lillo, Miguel Lillo 205, Tucumán, CP 4000, Argentina

## Abstract

**Background:**

The family Pteropodidae comprises bats commonly known as megabats or Old World fruit bats. Molecular phylogenetic studies of pteropodids have provided considerable insight into intrafamilial relationships, but these studies have included only a fraction of the extant diversity (a maximum of 26 out of the 46 currently recognized genera) and have failed to resolve deep relationships among internal clades. Here we readdress the systematics of pteropodids by applying a strategy to try to resolve ancient relationships within Pteropodidae, while providing further insight into subgroup membership, by 1) increasing the taxonomic sample to 42 genera; 2) increasing the number of characters (to >8,000 bp) and nuclear genomic representation; 3) minimizing missing data; 4) controlling for sequence bias; and 5) using appropriate data partitioning and models of sequence evolution.

**Results:**

Our analyses recovered six principal clades and one additional independent lineage (consisting of a single genus) within Pteropodidae. Reciprocal monophyly of these groups was highly supported and generally congruent among the different methods and datasets used. Likewise, most relationships within these principal clades were well resolved and statistically supported. Relationships among the 7 principal groups, however, were poorly supported in all analyses. This result could not be explained by any detectable systematic bias in the data or incongruence among loci. The SOWH test confirmed that basal branches' lengths were not different from zero, which points to closely-spaced cladogenesis as the most likely explanation for the poor resolution of the deep pteropodid relationships. Simulations suggest that an increase in the amount of sequence data is likely to solve this problem.

**Conclusions:**

The phylogenetic hypothesis generated here provides a robust framework for a revised cladistic classification of Pteropodidae into subfamilies and tribes and will greatly contribute to the understanding of character evolution and biogeography of pteropodids. The inability of our data to resolve the deepest relationships of the major pteropodid lineages suggests an explosive diversification soon after origin of the crown pteropodids. Several characteristics of pteropodids are consistent with this conclusion, including high species diversity, great morphological diversity, and presence of key innovations in relation to their sister group.

## Background

The family Pteropodidae comprises 186 currently recognized species commonly known as Old World fruit bats or megabats, and as such constitutes one of the largest families of the order Chiroptera (Mammalia) [1]. The name "megabats" is an abbreviation for Megachiroptera, and was coined in recognition of the large body size of some pteropodid species, which can reach over 1 kilogram in weight and have a wingspan of over 1.5 meters [2]. Pteropodids are almost exclusively phytophagous, feeding mostly on fruits, although some species are specialized for nectar feeding. As a group, pteropodids are primary dispersers of pollen and seeds in the Old World tropics [2]. They have a widespread distribution in Africa, the tropics of Asia, and Australia as well as occurring on many islands from the Indian Ocean to the Western Pacific Ocean, where some species are highly endangered and in risk of extinction [1,3]. Unlike other bat families, members of Pteropodidae do not use laryngeal echolocation, instead relying primarily on vision and olfaction to avoid obstacles and locate food sources.

Phylogenetic relationships of Pteropodidae have been the source of considerable debate. In the 1990s a controversy developed over whether or not Chiroptera was monophyletic or instead composed of two different evolutionary lineages (Megachiroptera and Microchiroptera) that achieved powered flight independently from origins within different parts of the mammalian family tree (see [4], and references cited therein). Comprehensive analyses of morphological and molecular data refuted this hypothesis and confirmed bat monophyly [5-8] but analyses of DNA sequence data revealed a surprise - some "microbats" were in fact more closely related to Pteropodidae than to the remaining Microchiroptera families [9,10]. This led to a revision of higher-level bat taxonomy that saw Megachiroptera and Microchiroptera discarded and two new groups recognized, Yinpterochiroptera (for Pteropodidae and its close relatives, echolocating bats of the superfamily Rhinolophoidea) and Yangochiroptera (for the remaining echolocating bats) [11].

All of these higher-level studies confirmed monophyly of Pteropodidae, but considerable confusion has remained concerning relationships within this clade. Contra traditional classifications (e.g., [12]), early molecular phylogenies of the group discovered that nectar-feeding megabats did not constitute a single monophyletic group, but instead various nectar-feeding genera were related to different fruit-feeding lineages (e.g., [8,13]). These relationships were formally recognized in the classification of Bergmans [14], who divided the group into six extant subfamilies and several tribes (Table 1). Subsequent phylogenetic studies of pteropodids have further questioned or strongly refuted commonly recognized groupings (subfamilies, tribes), and identified new, novel groupings of taxa [13,15-21]. The most comprehensive studies to date based on molecular sequence data included 26 genera and were based mostly on mitochondrial genes including 12S rRNA, 16S rRNA and the *Cytochrome b *gene plus a small sample of nuclear data (~400 bp of the oncogene *cmos*) [18,19]. Giannini and Simmons [19] confirmed the monophyly of Pteropodidae and of many higher-level taxa defined by Bergmans [14] (e.g., Pteropodini, Macroglossini, Dobsoniini, Epomophorinae, Epomophorini, Myonycterini, Cynopterinae). Nevertheless, support values for some nodes were low and resolution was poor in several parts of the tree. The weakest part of the phylogeny was one of the most critical parts -- the backbone, which ideally should show how the various higher-level groups are related to one another [19]. Basal relationships among subfamilies and tribes were discordant among different data treatments and generally received low statistical support. For this reason, questions still remain regarding relationships within and between major pteropodid clades.

**Table 1:** Bergmans (1997) classification of Family Pteropodidae

Subfamily	Tribe	Genera
Pteropodidae	Pteropodini	*Pteropus*, *Acerodon*, *Pteralopex*, *Styloctenium*, *Neopteryx*
	Macroglossini	*Macroglossus*, *Syconycteris*
	Notopterini	*Notopteris*, *Melonycteris*
Nyctimeninae		*Nyctimene*, *Paranyctimene*
Harpyionyterinae		*Harpyionycteris*
Rousettinae	Rousettini	*Rousettus*, *Eonycteris*, *Eidolon*
	Dobsoniini	*Dobsonia*, *Aproteles*
Epomophorinae	Epomophorini	*Epomophorus*, *Micropteropus*, *Hypsignathus*, *Epomops*, *Nanonycteris*
	Myonycterini	*Myonycteris*, *Lissonycteris*, *Megaloglossus*
	Scotonycterini	*Scotonycteris*, *Casinycteris*
	Plerotini	*Plerotes*
Cynopterinae		*Cynopterus*, *Ptenochirus*, *Megaerops*, *Dyacopterus*, *Balionycteris*, *Chironax*, *Thoopterus*, *Sphaerias*, *Aethalops*, *Penthetor*, *Latidens*, *Alionycteris*, *Otopteropus*, *Haplonycteris*

Lack of resolution along the backbone of a phylogenetic tree can result from sampling bias (or stochastic bias), systematic bias, or a combination of both [22]. Sampling bias occurs when a data set does not contain enough information to allow full resolution of taxon relationships, either due to inadequate taxonomic sampling or lack of phylogenetic signal in the sampled loci [23-26]. Both factors could have influenced previous phylogenetic analyses of megabats, which were based mostly on mitochondrial loci that may be saturated with substitutions at the tribal/subfamily level [27]. Incompleteness of taxonomic sampling, which may contribute to poor phylogenetic results particularly at higher taxonomic levels, clearly could have played a role in pteropodid analyses, as only 26 out of the 46 pteropodid genera currently recognized (57%) were represented in the largest, previous molecular studies. Another type of bias, known as systematic bias, is caused by non-phylogenetic noise in the sequences such as differences in base composition (causing unrelated taxa with similar base composition to erroneously cluster together) and/or substitution rate (causing long-branch attraction) (e.g. [28,29]). Because these types of variation violate the assumptions of most reconstruction methods, they are also potential causes for inaccuracy and poor resolution in phylogenetic trees [22,30,31]. Moreover, sampling bias and systematic bias may synergistically interact, compounding their effects on the outcome of phylogenetic inference [26]. None of these potential sources of systematic bias have been addressed in previous phylogenetic studies on megabats.

An alternative explanation for low resolution of relationships on a phylogenetic tree is that, instead of being a consequence of bias or methodological artifacts, it reflects the true evolutionary history of the group. Rapid diversification of a clade over a short period of time may cause such a phylogenetic pattern, as has been claimed for the origin of the mammalian [32,33] and avian orders [34,35]. If diversification took place quickly and long time ago, there may be little phylogenetic signal because slowly-evolving genes may not have accrued many changes (due to the short time span) while faster-evolving genes may have initially picked up changes, but these were then overwritten by subsequent sequence evolution. Also, short time interval between cladogenetic events may hinder complete lineage sorting, confounding relationships within that time frame [36]. Such trees, with very short internal branches connected to the root, are also known as star phylogenies (e.g. [37,38]).

Here we present a comprehensive study of the phylogenetic relationships among megabat genera based on a large DNA-sequence dataset. Our focus was on resolving tribal membership and relationships among subfamilies. We attempted to address potential sources of bias in the phylogenetic reconstruction of this group, including low number of informative characters, missing data, poor taxonomic sampling, and sequence bias. This was accomplished by obtaining new sequences for four nuclear loci, thus significantly increasing taxonomic sampling, and by filling in gaps in sequence data from mitochondrial loci that had been previously sequenced in megabats by collecting new data from the additional taxa available to us. The inclusion of additional taxa not only adds important information on variation but can also help break up long branches, thus improving phylogenetic accuracy and helping resolve clades that may remain ambiguous with smaller taxon samples [23,39-41]. The simultaneous analysis of several genes has the obvious advantage of increasing the number of phylogenetically informative characters and balancing stochastic errors [42-48], but can also reduce the effects of systematic bias that may affect individual gene partitions, especially when locus-specific substitution models are employed in a probabilistic framework [31,49-51].

To minimize the effects of various potential sources of error in our analyses, detailed phylogenetic analyses were conducted using tests for systematic sequence bias, different reconstruction methods, optimal data partitioning in maximum likelihood analyses, and topology comparisons based on data simulations. Our results defined 7 well-supported groups for a new, cladistic classification of pteropodids, in several points different from the last formal classification by Bergmans [14]. The most basal relationships within Pteropodidae, however, could not be fully resolved despite the increase in the amount of data and the use of careful phylogenetic analyses. This result could not be explained by any source of measurable sequence bias, pointing to a biological cause for the observed pattern.

## Results

### Sequence data statistics

To minimize the effects of missing data on tree resolution [52], we first focused on a dataset that included 51 pteropodid species for which at least four of the six sequence fragments (RAG1, RAG2, vWF, BRCA1, Cytb, and 12S16S) used in this study were available (dataset 1). The combined alignment of dataset 1 was 8181bp long, yielding 2504 parsimony informative sites. Details of sequence statistics of dataset 1 in the combined and per loci matrices are shown in Table 2. A saturation plot of the combined dataset (ingroup only, without taxa with missing one or more loci) did not show signs of substitution saturation (Additional file 1), which was confirmed by a statistical test for saturation (Iss = 0.645, Iss.cSym = 0.844, p < 0.0001) [53].

**Table 2:** Sequence statistics and maximum parsimony scores per gene and in the combined dataset 1

locus	RAG1	RAG2	VWF	BRCA1	*Cytb*	12S16S	dataset 1
alignment (bp)	1084	760	1231	1352	1140	2566	8181
invariable	851	567	811	848	592	1487	4648
pars. infor.	154	115	256	296	502	864	2504
CI MP^a^	0.536	0.582	0.516	0.748	0.210	0.287	0.334
RC MP^b^	0.357	0.410	0.322	0.605	0.077	0.138	0.165

^a ^Consistency Index. ^b ^Rescaled Consistency Index

Despite differences among genes and codon positions, we found relative homogeneity in GC content among pteropodid taxa (Additional file 2). The χ^2 ^test failed to detect significant differences in either entire genes or individual codon positions. We also checked for similarities and differences in GC content among the principal clades of Pteropodidae that could bias the resolution of the relationships among them (Additional file 2). Except for the 3^rd ^codon position of the Cytb locus, GC content showed little among clade variation. Rates of nucleotide substitution were also relatively homogeneous, with only 7 pairwise taxa comparisons showing significant differences. All these comparisons involved an outgroup taxon compared with an ingroup taxon; no significant rate differences could be detected within the ingroup (Pteropodidae). Borderline *p *values were found in some comparisons involving *Balionycteris maculata*, but not in other pairwise comparisons. This result suggests the long-branch attraction is not likely to have a major effect in our phylogenetic results.

### Phylogenetic analyses of individual genes

MP and ML analyses were run for each locus separately (ML trees are available in the Additional file 3). Most topological differences among resulting trees were seen in basal relationships, but none was supported by > 60% bootstrap, which suggests absence of incongruent phylogenetic signal among genes [54]. In a parsimony framework, the pairwise ILD tests did not detect significant pairwise incongruence. The same result was obtained with the likelihood based hierarchical test [55].

### Maximum parsimony analyses of the combined dataset

**Figure 1:**
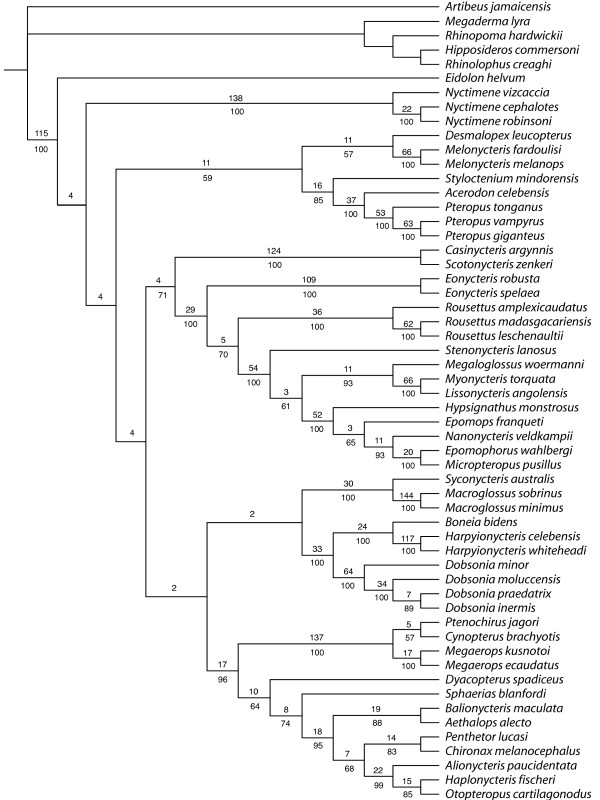
**Single most parsimonious tree recovered with dataset 1**. Tree scores are shown in Table 2. Numbers above branches are Bremer decay values and below branches are bootstrap percentages (when above 50%) obtained with 1000 replicates.

The maximum parsimony (MP) analysis of the combined dataset 1 recovered one most parsimonious tree with 15637 steps and consistency index of 0.334 (Figure 1). This tree showed a monophyletic Pteropodidae with six well-supported internal clades and one independent lineage. Some of these clades are congruent with previously proposed subfamilies, such as Cynopterinae [14], Harpyionycterinae [21], and Nyctimeninae and tribes, such as Pteropodini, Scotonycterini, Macroglossini, and Epomophorini [14]. Basal relationships among these seven main pteropodid groups, however, were resolved with only low support in both Bremer decay values (<5) and bootstrap precentages (<80%, Figure 1). To check whether variation in GC content in 3^rd ^codon position of the *Cytb *gene could be affecting the results, we reanalyzed the data eliminating this partition from the matrix (Additional file 4). The only difference in topology was that the African clade was the third pteropodid clade to diverge instead of Pteropodini as shown in Figure 1, but basal relationships had even lower support.

### ML analyses using optimal partition schemes

**Figure 2:**
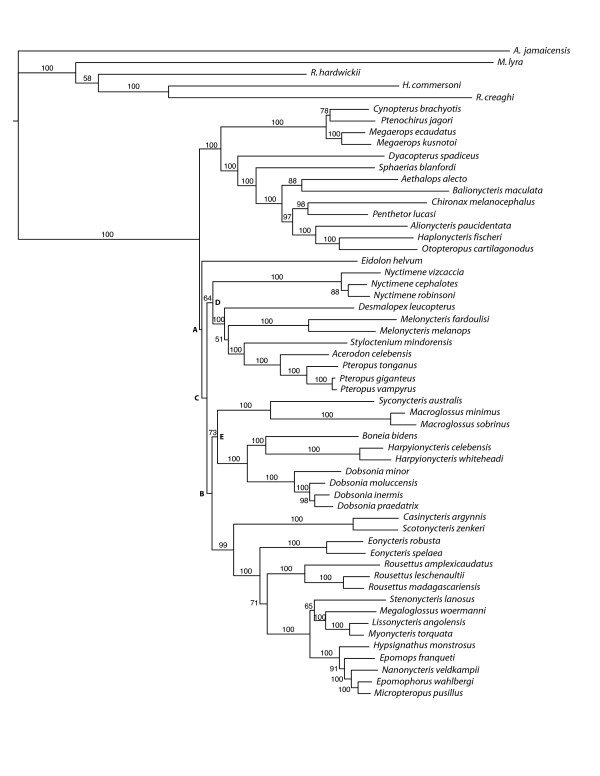
**Maximum likelihood tree obtained with dataset 1 and partition scheme 6**. Substitution models and parameters used are listed in the Additional file 6. Bootstrap values above 50% are shown next to branches.

To choose a partition scheme of the sequence data to be used in the maximum likelihood (ML) analyses, we compared several alternative schemes under the GTR model, based on their AIC/AICc and BIC scores (Table 3). The trees obtained with those different schemes recovered the same principal clades as the MP tree, only varying the relationships among them (Additional file 5). Given the small improvement in AIC and BIC scores in partition scheme 7 as compared to scheme 6, we analyzed both schemes under optimal substitution models for each partition. Optimal substitution models were selected using AICc for the different partitions in schemes 6 and 7 (Additional file 6). The analyses based on partition schemes 6 and 7 resulted in the same topology, very similar to ML topology B (obtained with scheme 6 and the GTR+Γ model applied across all partitions, Additional file 5), which we will call henceforth optimal ML tree (Figure 2). The only difference between these two topologies is the position of *Eidolon*: while in topology B *Eidolon *appears as the most basal branch, in the optimal ML tree it appears as the second most basal branch with cynopterines occupying the most basal position. Branch support was assessed using partition scheme 6, since this scheme had fewer parameters and comparable results in both likelihood scores and topology to those obtained with partition scheme 7. The basal relationships within Pteropodidae again had no statistical support. The principal clades and clades within those, however, received substantial support, with very few bootstrap values below 80% (Figure 2). Similarly to results in MP analyses, removal of the 3^rd ^codon position of the Cytb partition did not affect the results (Additional file 4).

**Table 3:** Partition schemes used in ML analyses of combined dataset 1

scheme	#^a^	partitions^b^	ln L	AIC/AICc	BIC	parameters	topology^c^
1	1	no partition	-94660.30	189554.6	190374.7	117	A
2	4	codon_1, codon_2, codon_3, 12S16S	-90704.00	181696	182705.4	144	A
3	5	nuclear_12, nuclear_3, Cytb_12, Cytb_3, 12S16S	-86566.50	173439	174511.4	153	B
4	6	RAG1, RAG2, vWF, BRCA1, Cytb, 12S16S	-90465.01	181254	182389.6	162	C
5	7	nuclear_1, nuclear_2, nuclear_3, Cytb_1, Cytb_2, Cytb_3, 12S16S	-86362.20	173066.3	174264.9	171	D
6	11	RAG1_12, RAG1_3, RAG2_12, RAG2_3, vWF_12, vWF_3, BRCA1_12, BRCA1_3, Cytb_12, Cytb_3, 12S16S	-85935.75	172296.3	173736.5	207	B
7	16	each codon position for each coding gene, 12S16S	-85683.00	171887.5	173637.8	252	E

^a ^Number of partitions. ^b ^Numbers after underscore represent codon position. ^c ^Topologies are illustrated in the Additional file 5.

### Topology comparisons

The principal Pteropodidae clades were well defined and supported by all the different phylogenetic analyses we conducted. However, there was considerable disagreement among analyses concerning relationships among these clades. To decide whether one particular arrangement could be justifiably preferred to others, we carried out a series of topology comparison tests. On a MP framework, we found the optimal ML tree (tree length = 15670) to be significantly worse than the MP tree (tree length = 15636) by both the KH (p = 0.007) and the TN (p = 0.006) tests. On the other side, neither test distinguished the MP tree from ML topology B (tree length = 15647). We did the same comparisons in a ML framework using the SH and the AU tests, but no significant likelihood differences were found among the different hypotheses.

### Tests for zero-length branches

**Figure 3:**
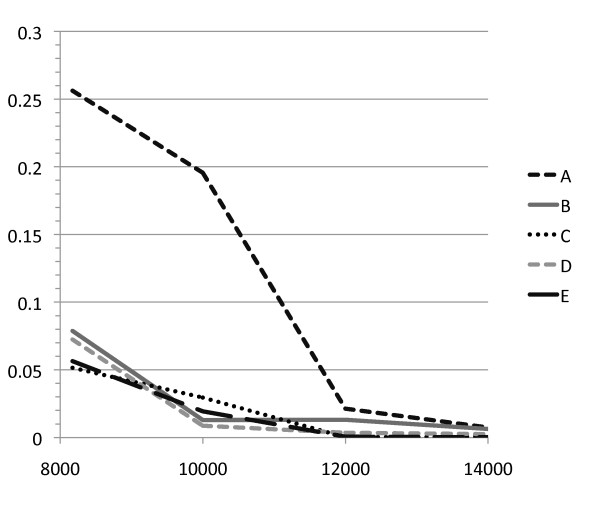
**P value of SH test versus length of simulated datasets**. SH tests were done between ML best trees and derived trees with a collapsed node for the original dataset (8174 bp) and three simulated datasets (10 kb, 12 kb, and 14 kb). The simulated datasets were obtained with the same nucleotide frequency and substitution parameters found in the original dataset. Nodes A, B, C, D, and E are labeled in Figure 2.

In all four loci tested, several basal branches could be simultaneously collapsed without significant likelihood differences between the collapsed and the best gene tree as compared to the null distribution obtained with simulations: four branches for RAG1, five for vWF, six for BRCA1, and five for 12S16S (Table 4). Analyzing the combined matrix, five basal branches could be separately collapsed without significantly changing tree likelihood according to the SH and the AU tests (at 1% significance level; Additional file 7). Nevertheless, only three of them could be simultaneously collapsed with the same result (only two nodes at 5% significance; Table 4). These results are in agreement with the idea that a substantial increase in the amount of data allows resolving splits that happen in a very short period of time. To determine how much sequence data would be necessary to resolve each of the basal nodes we simulated datasets of 10 kb, 12 kb, and 14 kb based on the best tree, using the same sequence parameters of the original combined dataset. The best trees obtained for the simulated datasets were then compared with trees derived from the best tree but with each of the basal nodes collapsed separately. Significant resolution of basal nodes as measured by pSH < 0.01 would be obtained with 10 kb for node D, 12 kb for nodes C and E, and 14 kb for nodes A and B (Figure 3, Figure 2).

**Table 4:** Results of tests for zero-length branches on gene trees

gene	collapsed nodes^a^	Dli simulations^b^	Dli main^c^
BRCA1	A+B+C+D+E+1	0.197 - 12.408	3.238
RAG1	A+C+D+E	0.054 - 14.313	10.61
vWF	A+B+C+D+E	0.893 - 13.801	5.479
12S16S	A+B+C+E+1	0.773 - 9.641	6.007
combined	A+B+C	0.043 - 5.656	4.496

^a ^Nodes are labeled on Figure 2 of the manuscript. ^b ^Range of the likelihood differences between collapsed tree and best tree obtained over 100 simulations, representing the null distribution for the main likelihood comparison. ^c ^Likelihood difference between the collapsed tree and the actual best tree.

### The position of additional genera

**Figure 4:**
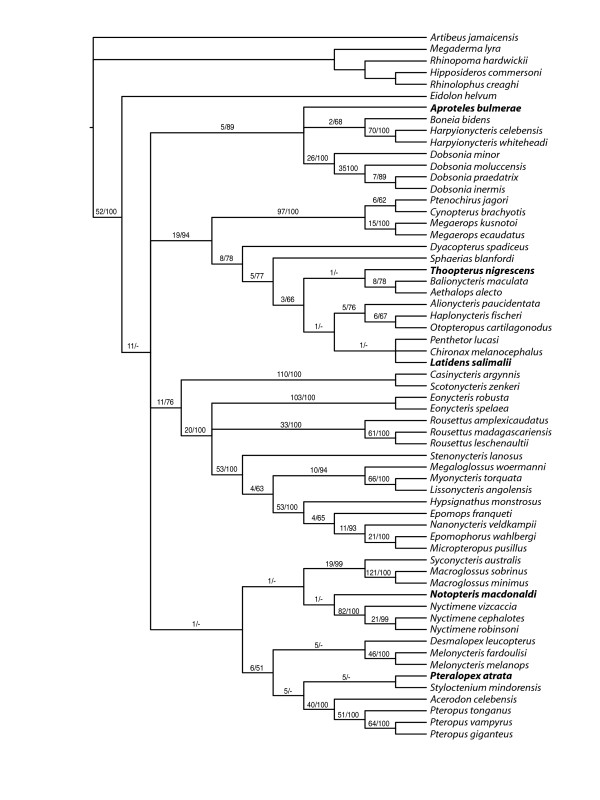
**Consensus of six most parsimonious trees obtained with dataset 2**. Numbers shown above branches refer to Bremer decay values (left) and bootstrap percentages (right).

**Figure 5:**
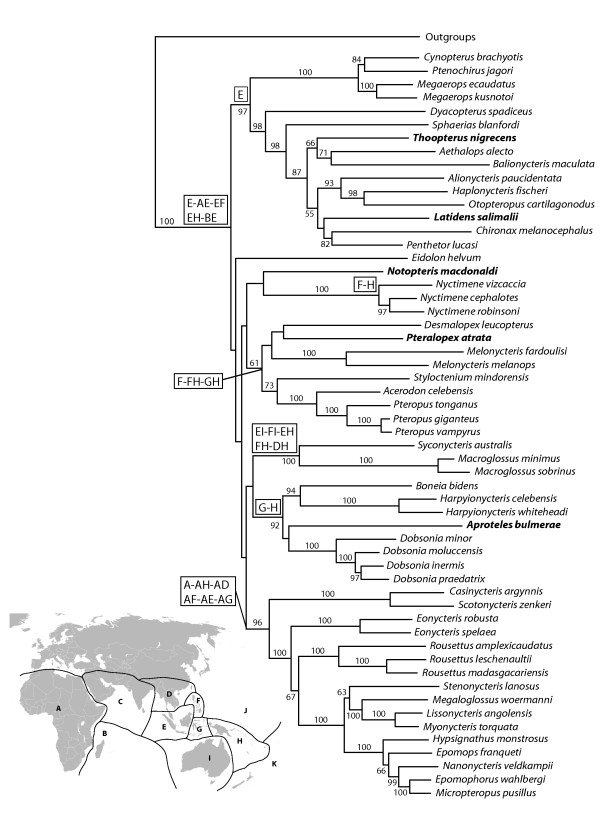
**ML tree obtained with dataset 2 and partition scheme 6**. Terminals in bold letters were represented in the data matrix by only two gene partitions. Bootstrap values greater than 50% are shown. Letters inside rectangles represent the inferred possible ancestral areas (labeled on the map insert and in the Additional file 9) for the referred clades.

Combined dataset 2 included five additional genera for which data were available for only one or two mitochondrial loci; the dataset thus includes 56 ingroup taxa and five outgroup taxa. The MP analysis of dataset 2 resulted in six equally parsimonious trees with 16389 steps. The consensus tree has again *Eidolon *as the most basal pteropodid and a polytomy including all other principal clades as recovered in our analyses of dataset 1 (Figure 4). Most of the extra genera included in dataset 2 fell in clades according to expectations based on the most current classification of Pteropodidae [14,21], with the exception of *Notopteris*. Most of the relationships involving the additional taxa, however, had low or no statistical support. To analyze dataset 2 using ML methods, we used partition scheme 6 and the same optimal models as in our analysis of dataset 1. The resulting tree showed very similar relationships for the additional taxa as compared to the MP tree (Figure 5). The main difference was in the close relationships of *Pteralopex*, although the principal clade in which it fell was the same.

### Biogeographic analysis

The objective of our biogeographic analyses was to infer ancestral areas for the main pteropodid clades that appeared consistently across analyses. Because of missing taxa, a thorough analysis of the biogeographic history of pteropodids would not be accurate. Thus, we used DIVA [56] to infer ancestral areas for the clade containing all the pteropodids plus the six principal clades. These results were plotted on the ML tree obtained for dataset 2 (Figure 5). New Guinea and Melanesia Islands (area H) appear as a possible ancestral area for most internal clades, with the only exception being cynopterines. The results for the Cynopterinae subfamily agree with our previous analyses, indicating that this clade had its origins most likely in the Sundaic region [27]. Another interesting result is the origin of the clade formed by African genera, *Rousettus*, and *Eonycteris*. Although different area combinations have similar probabilities of being the ancestral area of that group, the African continent does appear as one of them, while all other alternatives are area combinations that include Africa.

## Discussion

### Phylogenetic relationships and systematics of pteropodids

Here we present the most complete ever analysis of the evolutionary relationships of pteropodid bats using a number of reconstruction and statistical approaches. The phylogenetic trees presented here, independently of the reconstruction method employed or the partition analyzed (from individual genes to combined data), almost unanimously recovered six principal clades and one independent lineage (*Eidolon*), variously joined by versions of a poorly supported backbone. By contrast, relationships of genera within each of those principal clades were generally consistent and in agreement across the different analyses performed. Some of the principal pteropodid clades recovered in our trees are congruent with previously proposed subfamilies, such as Cynopterinae [14], Harpyionycterinae [21], and Nyctimeninae [14]. The other three main clades, represented by Macroglossini, Epomophorinae + Rousettini, and Pteropodini + *Melonycteris *(all groups sensu Bergmans [14]), were in disagreement with previous classifications into subfamilies. Some of these discrepancies had already been observed in previous studies, such as the clustering of Epomophorinae + Rousettini [19].

The phylogeny of the subfamily Cynopterinae was recently addressed by Almeida et al. [27]. The two major cynopterine clades recovered in that study also appeared as supported groups in all topologies recovered in our analyses, suggesting that these groupings are stable to varying taxonomic sampling and character data representation. Similarly, a recently recognized and expanded group of megabats, Harpyionycterinae (see [20,21]), was also recovered in this study. This heterogeneous group is formed by two clades, the dobsoniine or bare-backed bats (*Dobsonia *and *Aproteles*) and the harpy bats from the Philippines and Sulawesi. *Boneia bidens*, a bat formerly included as a subgenus of *Rousettus *(e.g., [1,14]) joined this clade as sister to *Harpyionycteris *as previously reported [21]. The Pteropodini and Macroglossini tribes were also recovered as major clades in our study, but not as sister taxa or close relatives (Figure 4) in a monophyletic subfamily Pteropodinae, as proposed by Bergmans [14]. In this study, Pteropodinae was recovered as a clade composed of one nectarivorous genus (*Melonycteris*) associated to flying foxes and related megabats (*Acerodon*, *Desmalopex*, *Mirimiri*, *Pteralopex*, *Pteropus*, and *Styloctenium*). The exclusion of two genera (*Mirimiri *and *Neopteryx*) and the lack of statistical support for some internal relationships claim for a more detailed study of the pteropodines.

One major clade, including rousettines (excluding *Boneia *as discussed earlier) and all African megabats (excluding *Eidolon *as discussed earlier) was recovered consistently and with high support across all analyses in this study. Versions of this clade, although differing somewhat in taxonomic sampling, have been consistently recovered since Hollar and Springer [13] first investigated pteropodid relationships using molecular methods [16,18,19]. This clade was originally highly controversial because the molecular data joined taxa from disparate traditionally recognized taxonomic groupings: rousettines (*Eonycteris*, *Rousettus*, and *Stenonycteris*) and epomophorines (the remainder of the African, except for *Eidolon*) sensu Bergmans [14]. Giannini and Simmons [19], however, demonstrated morphological support for this now expanded "African clade". It is noteworthy that two other African genera, *Scotonycteris *and *Casinycteris*, included in the Epomophorinae subfamily by Bergmans [14] and here for the first time sampled in a molecular phylogenetic study, appeared as sister to that clade in our analyses. This finding has important biogeographic implications, pointing to an African origin of this group as shown in the results of the biogeographic analysis.

*Eidolon *is an unusual taxon that was the single megabat genus not linked to any other genera in a major clade. To some extent, this is a somewhat unsurprising result as affinities of *Eidolon *have always been contentious; however, it is remarkable that the observed placement of *Eidolon *does not seem to be an artifact from primary data since no significant codon bias nor differences in evolutionary rates could explain this result. Morphology tends to support an association of *Eidolon *and other large megabats in the pteropodine clade [19]. A clade formed by *Eidolon *+ Pteropodini, however, was not represented in any of the trees obtained with the combined dataset. This result suggests that the non-overlapping distribution of *Eidolon *versus pteropodines in continental Africa may have ecological and biogeographic rather than phylogenetic origin. *Eidolon *was included in the Rousettinae subfamily by Bergmans [14], but our results strongly suggest it should be in a separate subfamily by itself. It is possible that the Melanesian genus *Notopteris *represents another case of independent lineage, as suggested by the lack of statistical support for its relationship with other pteropodid genera. As only mitochondrial sequences were available for this genus, additional data will be required to resolve the affinities of *Notopteris*.

Despite general lack of agreement among partitions and methods of analysis and statistical support for the relationships of the principal pteropodid clades, two groupings seem to be slightly favored. These two groupings received more than 50% bootstrap support in the optimal ML tree and appeared in a few other recovered trees. One is the clustering of Macroglossini and Harpyionycterinae as sister clades also obtained in MP tree and several of the suboptimal ML trees (Figure 4), with maximum bootstrap support of 73%. The other is the clade formed by Nyctimeninae and Pteropodini, which received 64% bootstrap support in the optimal ML tree and was also recovered in other ML trees (ML topologies B, C, D - Additional file 5), but did not appear in the MP tree.

### Basal polytomy

Evolutionary relationships that cannot be resolved in a phylogenetic analysis may represent a soft or a hard polytomy. A soft polytomy is the result of analytical bias, while a hard polytomy illustrates biological phenomena such as an explosive radiation. Hard polytomies are so called because can only be broken with a large amount of data and careful analyses. Before indentifying a hard polytomy it is necessary first to eliminate possible bias that could cause a soft polytomy.

We were able to assemble a matrix with a wide genera representation and relatively little missing data. The concatenated matrix of six loci showed no signs of substitution saturation, and contained enough phylogenetic signal to resolve a strongly supported monophyletic Pteropodidae, the superfamily Rhinolophoidea *sensu *Teeling et al. [57], and most ingroup relationships (37 out of 49) with bootstrap > 96% (43 with bootstrap > 70%). Detailed characterization of the data did not uncover important systematic sequence bias that could blur phylogenetic signal [58]-- megabats were shown to be relatively homogeneous in nucleotide composition at most partitions and in evolutionary rates, and no significant conflicting phylogenetic signal was detected among the different loci used. Moreover, phylogenetic analyses under the ML framework using specific substitution models for different partitions of the data most likely accounted for any minor sequence bias that could have affected the analyses [31,50,51,54,59].

Instead of having conflicting signal, the different loci used in this study agreed in a general lack of resolution at the base of the pteropodid tree. The results of the SOWH test support that at least some of the basal (inter subfamilies) relationships have zero-length branches in one or more gene partitions, some of which occurring across all partitions. As expected, an increase in the amount of data (i.e. the combined dataset) decreased the number of basal branches that could be simultaneously collapsed without affecting likelihood scores. Simulations of larger datasets suggest that the addition of about 6 kb to the Pteropodidae combined matrix used here would probably allow to resolve all basal relationships of the family. This result is similar to that obtained in the analyses of the relationships among bird orders. After being shown to represent a hard polytomy [34], the intraordinal relationships of Neoaves could finally be resolved with a 32 kb dataset [35]. Because the radiation of Neoaves is much older than that of Pteropodidae, it is expected that that group require more data for phylogenetic resolution. As previously suggested, the older the radiation, the greater the effect of rapid diversification on phylogenetic resolution [60].

### Explosive radiation of megabats?

Lack of phylogenetic resolution (hard polytomy) even when a considerable amount of data is used has been interpreted as evidence of closely spaced cladogenetic events [32,33,35,60,61]. Pteropodidae has apparently been distinct from other bat lineages since at least the early Eocene [10,62] but the crown group is believed to be of more recent origin. Estimates for the beginning of crown group divergence range between 31 and 20 million years (My) [10,27]. Using an estimate of 26 My for the pteropodid radiation and the substitution rates obtained with our combined dataset, the first three cladogenetic events of Pteropodidae are estimated to have occurred within approximately 0.5 My.

The results of our phylogenetic analysis, therefore, suggest that pteropodids experienced an explosive radiation that generated all main lineages representing its extant diversity. Although a more focused analysis would be necessary to fully evaluate this hypothesis, some characteristics of the family Pteropodidae are consistent with the idea that it experienced an explosive radiation. Explosive radiations are usually associated with high taxonomic diversity [63] and Pteropodidae is in fact one of the most diverse bat families. Among the 20 bat families currently recognized, Pteropodidae ranks second in both genus and species diversity with over 45 genera and over 180 species [1,64].

Explosive radiations can be caused by demographic factors, intrinsic evolutionary rates, ecological adaptation, or a combination of any or all of these factors [65]. When an explosive radiation is accompanied by ecological adaptation (adaptive radiation), it often involves the evolution of novel characters (key innovation) [63,65,66]. Pteropodidae exhibits numerous innovations when compared to their closest relatives (Rhinolophoidea and Yangochiroptera), including primary phytophagy and predominance of visual over acoustic orientation (for an extensive list of differences between megabats and microbats see [67]). Also in accordance with ecological adaptation as a drive to diversification is the marked morphological diversity of megabats, such as the high variance in body size, as compared to the other bat families [68] and the independent evolution of nectarivorous habits and associated morphological adaptations in several of the pteropodid clades. Among the demographic causes of explosive radiations are small population sizes (favoring differentiation through genetic drift) and/or the existence of isolated peripheral populations. Given the flight power of megabats and their geographic distribution on (often isolated) islands, colonization of underpopulated areas and the existence isolated peripheral populations could both have contributed for an explosive radiation of megabats.

## Conclusions

Our phylogenetic analyses identified six principal clades and one additional independent lineage within Pteropodidae. This result points to the need for a new formal classification of the family based on monophyletic units. The trees presented here are the most complete ever for the family in terms of genera representation, and are robust in terms of providing statistical support for pteropodid relationships. They thus provide a sound phylogenetic framework for the study of the morphologic, ecologic, and behavioral evolution within this highly diverse and divergent bat family. In contrast with the high statistical support obtained for the major pteropodid groups and subordinate clades, relationships among the seven principal clades were largely unresolved. Congruence in this aspect among different gene trees and the results of simulations and the SOWH test suggest that crown pteropodids experienced an explosive radiation soon after their origin. To further evaluate the hypothesis of an explosive radiation of megabats and determine the potential processes involved will require a number of additional analyses including estimates of divergence times, estimates of diversification rates, and comparisons with other mammalian families with similar divergence times. A complete genus-level taxonomic sampling along with complete locus representation will be important in these future analyses.

## Methods

### Sampling

The effect of missing data on phylogenetic estimation is still a matter of controversy [52,69-72]. Our preliminary analyses using data partitions of the concatenated gene matrix and maximum likelihood searches showed reconstruction problems when whole partitions were missing for certain taxa. Accordingly, in order to minimize any possible effects of missing data, we generated two different data matrices for phylogenetic analyses. In the first matrix (combined dataset 1), our goal was to minimize missing data while including as much as possible of the generic diversity of Pteropodidae. This matrix included 51 pteropodid species, representing 37 of the 46 pteropodid genera (Additional file 8). Among these 51 ingroup samples, 50 had all sequences determined experimentally by us from tissue samples donated by several institutions and individuals. Sequences of the remaining species were obtained from the Genbank (NCBI-NIH). In this first matrix, 44 of the ingroup taxa were represented by all eight genes used in the analyses, four taxa had one missing gene, and one taxon had two missing genes. Some of the sequences obtained by us have already been published [20,21,27,73].

A second matrix (dataset 2) was built to include five additional pteropodid genera for which only a few sequences are available (Additional file 8). These genera were mostly represented by two mitochondrial genes, usually the ribosomal genes 12S and 16S. Most of these sequences were obtained from the Genbank, except for those of the genus *Latidens*, which we sequenced ourselves. Dataset 2 included all currently recognized pteropodid genera except four taxa for which no DNA sequence is available (*Paranyctimene*, *Mirimiri*, *Neopteryx*, and *Plerotes*) [1,74].

As outgroups, we used sequences from Genbank of five non-pteropodid bats. Four of these, *Rhinopoma hardwickii*, *Hipposideros commersoni*, *Megaderma lyra*, and *Rhinolophus creaghi*, belong to the yinpterochiropteran superfamily Rhinolophoidea, which is widely accepted as the sister group of Pteropodidae [9,10]. The fifth outgroup species, *Artibeus jamaicensis*, represents the other chiropteran suborder, Yangochiroptera.

### Molecular methods

Eight genes were sequenced for this study, including both nuclear and mitochondrial loci. The four nuclear gene regions included the exon 28 of the von Willebrand Factor gene (*vWF*, 1230 bp), partial Recombination Activating Gene 1 (*RAG1*, 1084 bp), partial Recombination Activating Gene 2 (*RAG2*, 760 bp), and partial Breast Cancer 1 gene (*BRCA1*, about 1370 bp). These genes have been used to reconstruct the phylogeny of the Chiroptera families and were able to resolve most interfamilial relationships [9]. Besides, these genes have been successfully used to resolve relationships at subfamily and genus levels in diverse groups of bats [21,27,73,75]. The four mitochondrial genes were sequenced in two fragments: one containing the complete sequence of the Cytochrome b gene (*Cytb*, 1140 bp) and another including partial rRNA 12S gene (1069 bp), the valine tRNA gene, and partial rRNA 16S gene (1330 bp), totaling about 2550 bp. The latter fragment has also proven highly informative at the familial level in Chiroptera [9]. The combined sequence set encompassed a total of 8181 bp of aligned nucleotides (including indels). Individual ingroup samples in dataset 1 had concatenated sequences ranging from 6011 bp to 8025 bp in length.

Total DNA was obtained from preserved tissue samples with the DNeasy tissue kit (QIAGEN). PCR amplification was carried out using previously published primers (*RAG1 *and *RAG2*: [9]; *vWF*: [76]; *Cytb*: [77]; 12S: [78]; 16S: [16,78]). New primers were designed for the 16S gene: 12l-f (AGAGGAGAYAAGTCGTAMCAAG), 16u-f (AGCCAYCAATTRAGAAAGC), 16q-r (GTTTGCCGAGTTCCTTTTAC), and 16k-r (ATAGATAGAAACCGACCTGGA); and the *BRCA1 *gene: BRCA1-f2 (AACAGATGGGTTGAAACTAAGG), BRCA1-f3 (AGGYGATTATGTTCAGAAGAAG), BRCA1-r2 (GAAGGCTAGGATTGACAAACTC), and BRCA1-r4 (ATTTAATTCTAGTTCCAYATTGC). Additional sequencing primers were also used for *vWF *, *RAG1 *[21] and 12S/valine-tRNA [27]. All sequences were obtained with an automated ABI 3730XL sequencer. Sequence editing and prealignment were done with the Sequencher 4.2 software (Gene Codes). Genbank accession numbers and voucher information for taxa included in this study are provided in the in the Additional file 8.

### Sequence statistics

Alignments were done using the program MAFFT [79] using the default costs for gaps (gap opening penalty = 1.53; gap extension penalty = 0.123). Among the protein coding genes, only *BRCA1 *had indels, all of which were in frame. Gap positioning was adjusted to match amino acid codon positions with MacClade 4.08 [80]. The fragment containing 12S-valine-tRNA/16S also contained several indels. This last fragment was treated as a single partition in our dataset, which totaled six gene partitions: RAG1, RAG2, vWF, BRCA1, Cytb, and 12S/val-tRNA/16S (hereafter "12S16S"). Conflicting phylogenetic signal among partitions was checked using the incongruence-length difference test (ILD) [81] as implemented in PAUP* 4.10 b [82], running 500 searches with random stepwise addition and 10 replicates per search. The ILD test has been criticized for being sensitive to both type I and type II errors [83,84]. As an alternative test for incongruence, we used the script concartepillar.py which employs a hierarchical clustering method and likelihood-ratio tests to identify pairs of loci that have incongruent phylogenetic signal [55]. In these tests we excluded taxa for which one or more loci were not available, so that each alignment had the same set of taxa.

Substitution saturation in the combined dataset was checked by plotting number of transition and transversions as a function of GTR distances. Additionally, we used a saturation test [53] implemented in the program DAMBE [85]. Base composition bias among taxa was analyzed for each locus including all codon positions and for each codon position separately using the χ^2 ^test implemented in PAUP* 4.10 b [82]. Pairwise relative rate tests were done using HyPhy [86]. In all multiple tests, significance was corrected for multiple testing using the sequential Bonferroni criterion.

### Phylogenetic analyses

Phylogenetic inferences were done using maximum parsimony (MP) and maximum likelihood (ML). The MP searches were run on PAUP* 4.10 b [82], with 1000 random sequence additions followed by tree bisection reconnection branch swapping (TBR). Gaps were treated as missing data. Statistical support for clades were obtained with non-parametric bootstrap using PAUP* 4.10 b and Bremer decay values using TreeRot v.3 [87].

ML analyses were carried out with the program TreeFinder [88]. For the combined dataset 1, we first tried several partitioning schemes and compared their outcomes to determine the optimal scheme. Seven different partition schemes based on gene and codon position were tested, from one that separates all genes and codon positions, totaling 16 partitions, to non-partitioning of the data (analyzing it as one single partition). In these analyses, the GTR model was generally applied, with partition-wide estimates of the rate parameters by maximum likelihood optimization and empirical nucleotide frequency parameters. The performances of the different partition schemes were evaluated by comparing the likelihood, AIC (Akaike Information Criterion) [89,90], and BIC (Bayesian Information Criterion) [91] values of the resulting trees. AICc was used instead of AIC in cases where the ratio of the number of parameters to the number of bases was equal or less than 40. AIC and BIC correct the likelihood of a model for the number of the parameters, penalizing overparameterization [92]. The partition schemes with better scores (lower AIC and BIC values) were then used in ML analysis with optimal substitution models for each partition. Selection of partition-wide substitution models was done in Treefinder, using AIC. Statistical support of branches was obtained with 500 replicas of partition-wise bootstrap.

Alternative topologies were compared in a ML framework using the Shimodaira-Hasegawa (SH) [93] and the Approximately Unbiased (AU) [94] tests as implemented in TreeFinder. In the MP framework, we used the program PAUP* to run the Kishino-Hasegawa (KH) and the Templeton non-parametric tests [95,96].

### Zero-branch length simulations

To test whether basal, non-supported branches had lengths significantly different from zero in the individual gene and combined dataset trees, we compared the best tree found for that dataset with trees identical to the best tree but with unsupported branches collapsed. For that we employed the SH and the AU tests using TreeFinder. To access whether multiple nodes were involved in a polytomy at the base of the Pteropodidae tree, we used a similar approach to that proposed by Poe and Chubb [34]. Briefly, for each best ML gene tree, basal branches were collapsed one by one simultaneously (up to 6), and the likelihood of these collapsed trees were compared with the likelihood of the best tree. Because typical likelihood comparison tests should not be employed when multiple branches are collapsed at the same time [97], we created null distributions for the test statistic (the difference in likelihood between the best and the collapsed tree) using simulated datasets as in the SOWH test [98]. Sequences were simulated based on trees with collapsed branches using the same evolutionary model and substitution parameters obtained for the original dataset with the program *evolver *of PAML v.4.4 [99,100]. For each of the 100 simulated matrices, we obtained the best tree over 10 independent runs using RAxML v7.2.6 [101] and calculated the difference in likelihood of this tree to that of the collapsed tree (used to simulate the sequences) using *baseml *(PAML v4.4). In this way we obtained a null distribution of 100 likelihood differences between best and collapsed tree (the null hypothesis tree). In the analyses involving simulations, only ingroup species for which all genes were sequenced (44 species) were kept in the matrix and the GTR+Γ model was generally employed. These analyses were not done for RAG2, due the low resolution of its gene tree (Additional file 3, Figure S4), and Cytb, because of the large difference in substitution rates between the 3^rd ^codon position and the other positions (Additional file 6).

### Biogeographic analysis

The biogeographic history of the major pteropodid clades was analyzed using DIVA (Dispersal-Vicariance Analysis) [56,102]. Geographic distribution of the pteropodid genera were obtained in Simmons [1] and double checked with updated information from the http://gis.miiz.waw.pl/webapps/thebats/iucn/ website. All species of the same genera were assigned the same distribution area, except for *Roussetus*. To remark that the most basal *Rousettus *in our tree (*R. amplexicaudatus*) inhabits the Asian continent, we assigned only its own area to this species, while to the other two *Rousettus *species (*R. leschenaultii *and *R. madagascariensis*) we assigned all areas occupied by species of this genus. The area units and the geographic distributions of genera used in the DIVA are listed in the Additional file 9 (Tables S5 and S6). DIVA was based on the ML tree obtained for dataset 2 (all available pteropodid taxa), using alternatively *maxarea=*2 or 3. Results obtained with *maxarea *= 3 option in general contained all the areas obtained with *maxarea *= 2 plus 3-area combinations of those areas. Hence, we show here only the results obtained with *maxarea *= 2.

## Authors' contributions

FCA did the laboratory work, analyzed the data, and drafted the paper. NPG and NBS obtained necessary tissue samples from institutions and individuals. All authors participated in the design of the study, contributed to the writing of the paper, and approved its final version.

## Supplementary Material

Additional file 1 **Saturation plot of the combined dataset 1**. Figure S1 represents a saturation plot of the combined dataset 1 (with the exclusion of taxa missing one or more loci) based on GTR distances. Click here for file

Additional file 2 **GC content**. Table S1 shows GC content statistics per gene and codon position, across all taxa and within main pteropodid clades. Click here for file

Additional file 3 **ML gene trees**. The file contains Figures S2 through S7 illustrating gene trees obtained by maximum likelihood with each individual gene partition analyzed here: RAG1, RAG2, vWF, BRCA1, 12S16S and Cytb. Click here for file

Additional file 4 **Trees obtained with the exclusion of 3^rd ^codon positions of Cytb^rd^**. Figure S8 illustrates the MP tree with Bremer decay values and Figure S9 illustrates the ML bootstrap tree. Click here for file

Additional file 5 **ML topologies obtained with different data partition schemes**. Figure S9 illustrating resumed ML trees obtained with dataset 1 under alternative partition schemes as described in Table 4 (main text). Click here for file

Additional file 6 **Substitution models and parameters**. Table S2 listing optimal substitution models and parameters for each partition under scheme 6. Click here for file

Additional file 7 **P values of tests for zero-length branches**. Table S3 showing P values of the SH and the AU tests for zero-length branches based on the combined dataset 1 for each main pteropodid node labeled on Figure 2 (main text). Click here for file

Additional file 8 **Sequences used in this study**. List of samples with Genbank accession numbers of sequences used in this study (Table S4). Click here for file

Additional file 9 **Areas and generic distributions used in the biogeography analysis**. Table S5 lists area units and Table S6 the generic distribution in those areas as used in the biogeography analysis. Click here for file
