# Supplementary material for: Evolutionary relationships of the old world fruit bats (Chiroptera, Pteropodidae): Another star phylogeny?
Source: BMC Evol Biol. 2011 Sep 30;11:281. doi: 10.1186/1471-2148-11-281 (PMC3199269; doi:10.1186/1471-2148-11-281)
Supplement: Additional file 1 — Saturation plot of the combined dataset 1. Figure S1 represents a saturation plot of the combined dataset 1 (with the exclusion of taxa missing one or more loci) based on GTR distances. [file 1471-2148-11-281-S1.PDF]

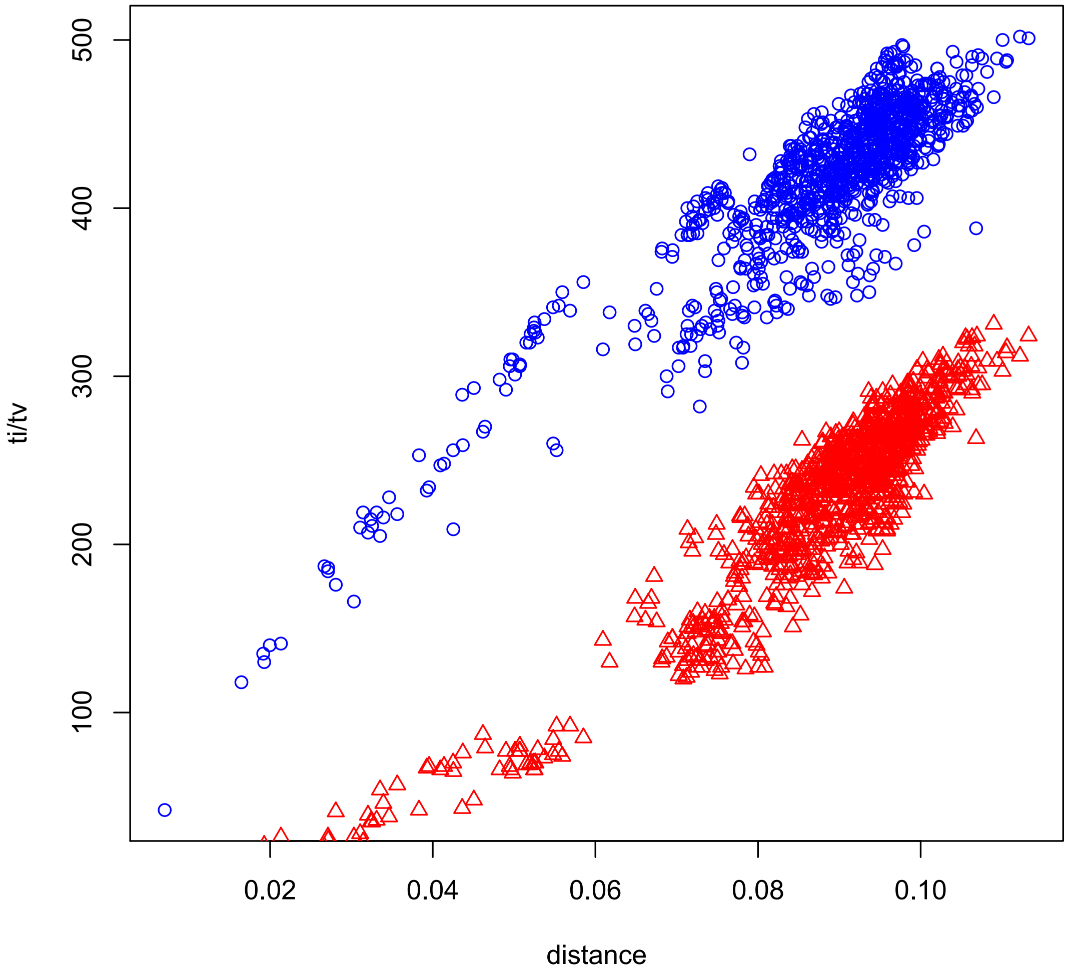

Figure S1. Saturation plot of the combined dataset 1 based on GTR distance, with the exclusion of taxa missing one or more loci. Transitions are represented by blue circles and transversions by red triangles.
