# Supplementary material for: Evolutionary relationships of the old world fruit bats (Chiroptera, Pteropodidae): Another star phylogeny?
Source: BMC Evol Biol. 2011 Sep 30;11:281. doi: 10.1186/1471-2148-11-281 (PMC3199269; doi:10.1186/1471-2148-11-281)
Supplement: Additional file 2 — GC content. Table S1 shows GC content statistics per gene and codon position, across all taxa and within main pteropodid clades. [file 1471-2148-11-281-S2.PDF]

Table S1. GC content per gene and codon position.

gene	codon	across all taxa				averaged within major clade							
		max	min	average	var(%)	A	C	E	H	M	N	P	var (%)
RAG1	1	52.4	50.7	51.7	3.3	51.9	51.5	51.5	52.0	51.6	51.8	51.2	1.6
RAG1	2	38.2	37.3	37.9	2.5	37.9	38.0	37.9	37.7	37.9	37.6	37.9	1.0
RAG1	3	63.3	58.3	60.8	8.2	61.6	60.7	59.4	62.1	59.6	59.7	59.3	4.7
BRCA1	1	46.5	42.6	44.6	8.9	45.3	44.3	44.9	44.1	44.0	44.2	44.2	3.1
BRCA1	2	39.9	37.1	38.5	7.4	38.6	38.6	38.8	39.1	38.3	37.3	38.0	4.5
BRCA1	3	29.1	25.7	27.2	**12.3**	27.1	27.0	27.5	26.9	27.3	27.8	27.8	3.3
vWF	1	67.0	62.6	64.7	6.8	64.7	64.4	63.9	64.5	65.1	66.8	64.6	4.5
vWF	2	39.3	36.3	38.2	7.9	38.5	37.7	37.0	38.5	38.6	38.4	37.9	4.2
vWF	3	88.4	79.5	84.6	**10.5**	85.6	84.3	79.5	84.4	82.8	87.6	83.4	9.7
RAG2	1	51.4	49.0	50.2	4.8	50.0	50.5	50.4	50.5	50.2	50.3	49.9	1.2
RAG2	2	41.0	39.0	40.1	5.0	40.5	40.0	39.8	39.8	39.8	39.3	40.2	3.1
RAG2	3	48.7	42.4	45.6	**13.8**	45.2	45.8	45.5	47.1	46.0	45.2	44.6	5.6
12S16S	na	42.4	39.0	40.6	8.4	41.1	40.6	42.2	40.0	39.7	40.2	40.3	6.2
Cytb	1	53.2	47.1	50.5	**12.1**	50.3	51.0	51.3	51.9	49.7	50.2	49.3	5.2
Cytb	2	38.7	36.9	37.8	4.8	37.9	37.5	38.2	38.2	38.0	37.6	37.5	1.9
Cytb	3	50.5	34.8	42.8	**36.7**	44.3	43.5	50.2	40.8	38.4	42.3	41.3	**27.6**

NOTE - Clades labels: A = African clade, C = Cynopterinae, E = *Eidolon helvum*, H = Harpyionycterinae, M = Macroglossini, N = *Nyctimene*, P = Pteropodini
