# Supplementary material for: Evolutionary relationships of the old world fruit bats (Chiroptera, Pteropodidae): Another star phylogeny?
Source: BMC Evol Biol. 2011 Sep 30;11:281. doi: 10.1186/1471-2148-11-281 (PMC3199269; doi:10.1186/1471-2148-11-281)
Supplement: Additional file 3 — ML gene trees. The file contains Figures S2 through S7 illustrating gene trees obtained by maximum likelihood with each individual gene partition analyzed here: RAG1, RAG2, vWF, BRCA1, 12S16S and Cytb. [file 1471-2148-11-281-S3.PDF]

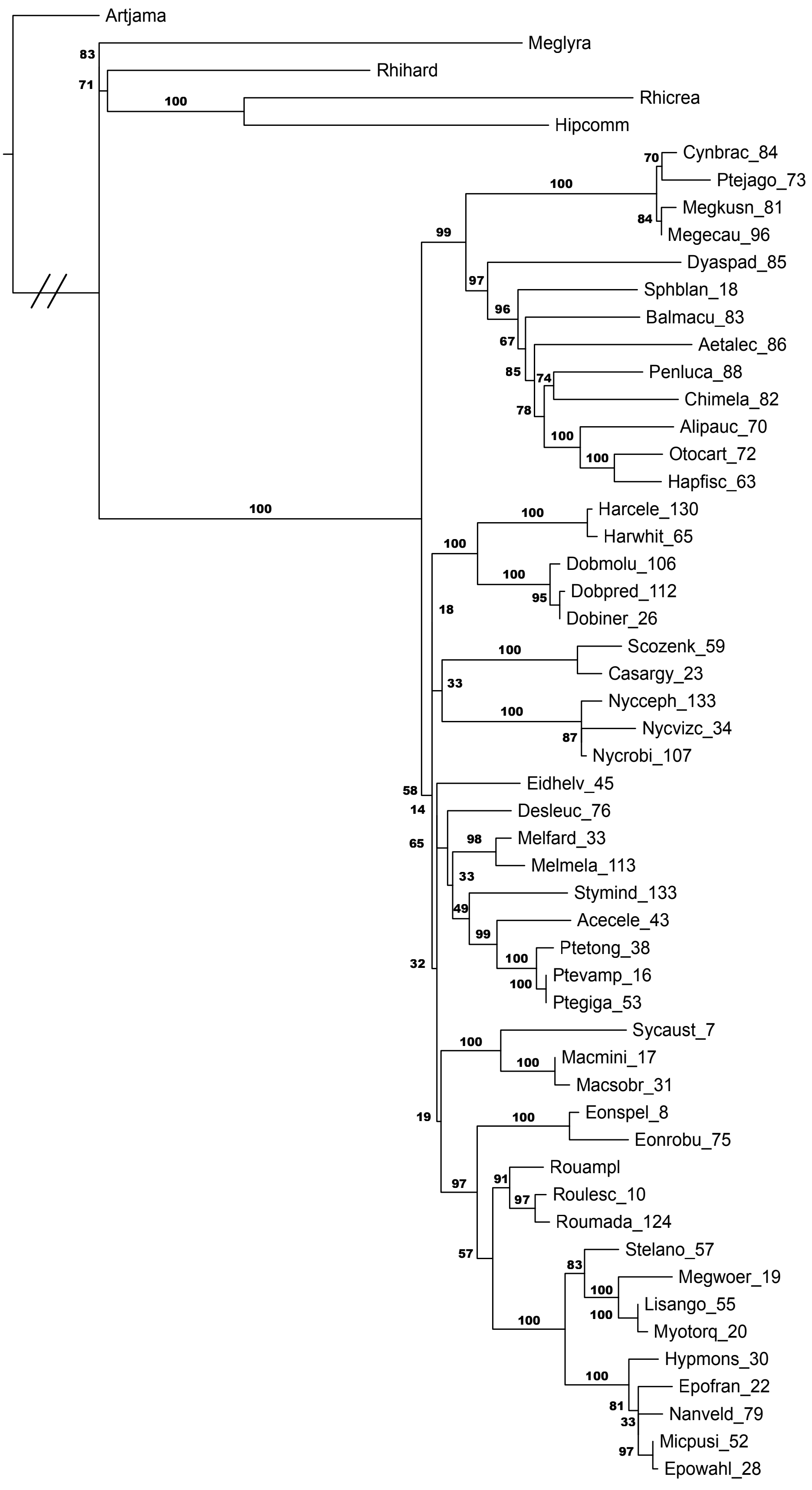

Figure S2. BRCA1 ML gene tree with bootstrap percentages.

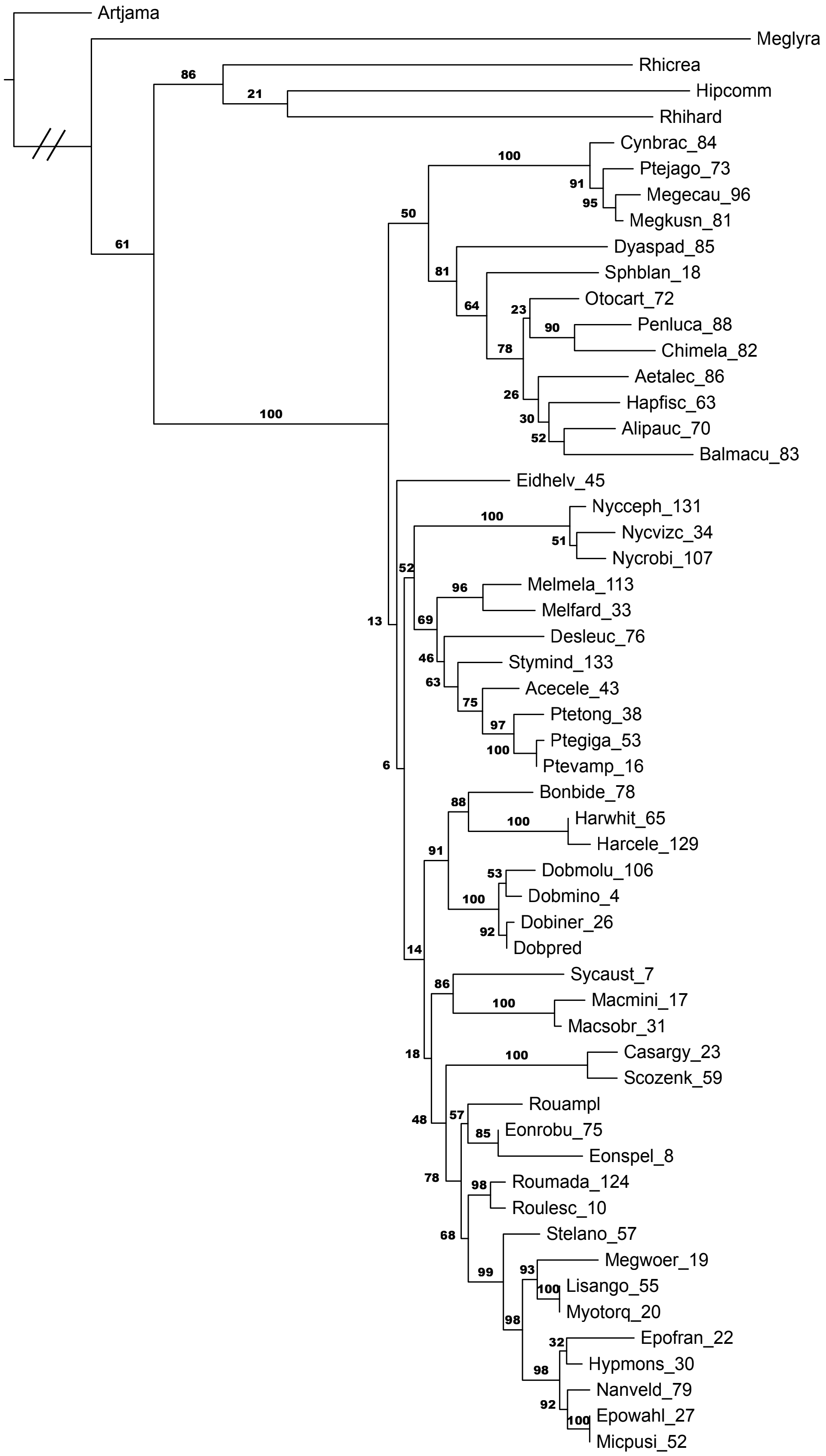

Figure S3. RAG1 ML gene tree with bootstrap percentages.

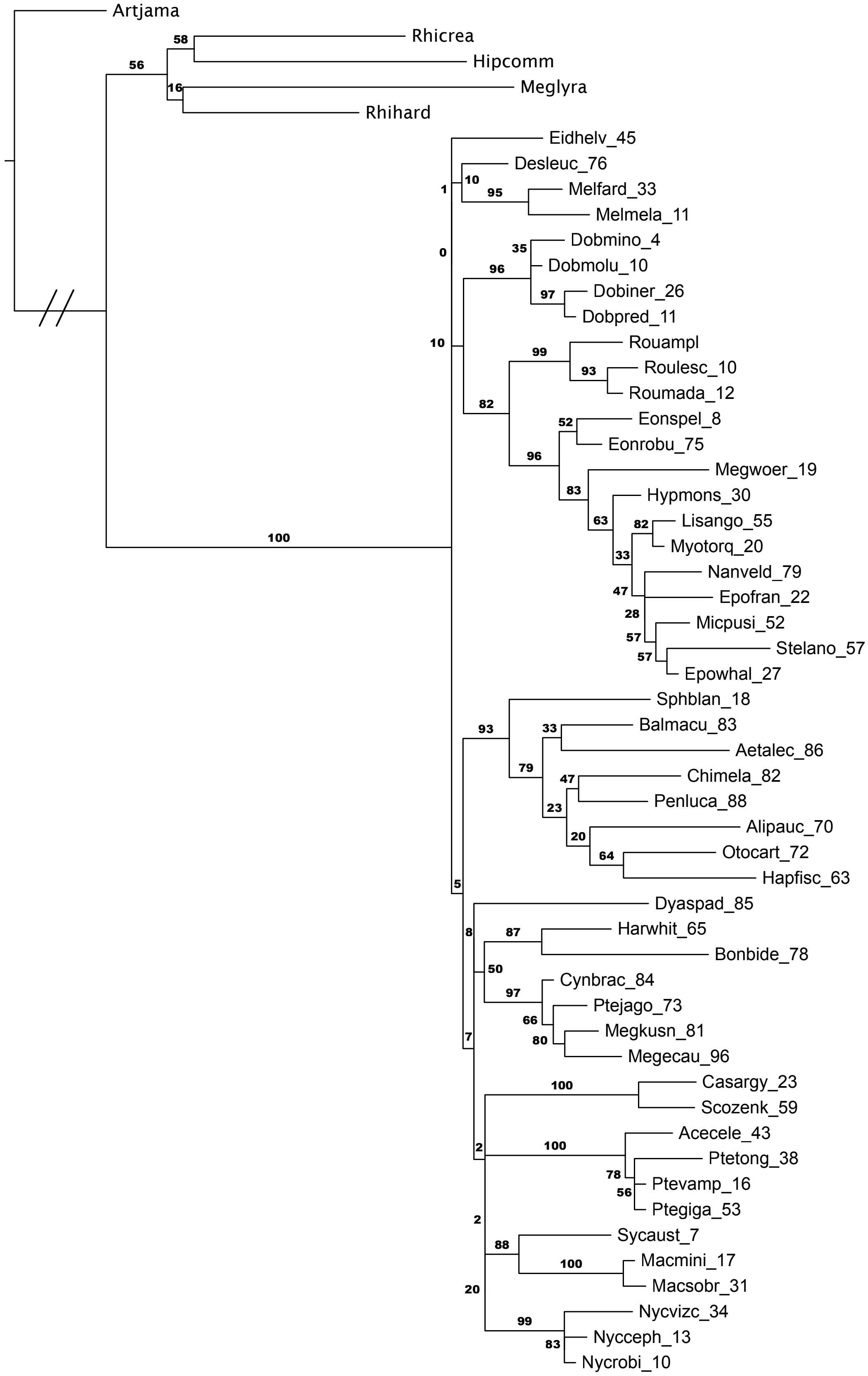

Figure S4. RAG2 ML gene tree with bootstrap percentages.

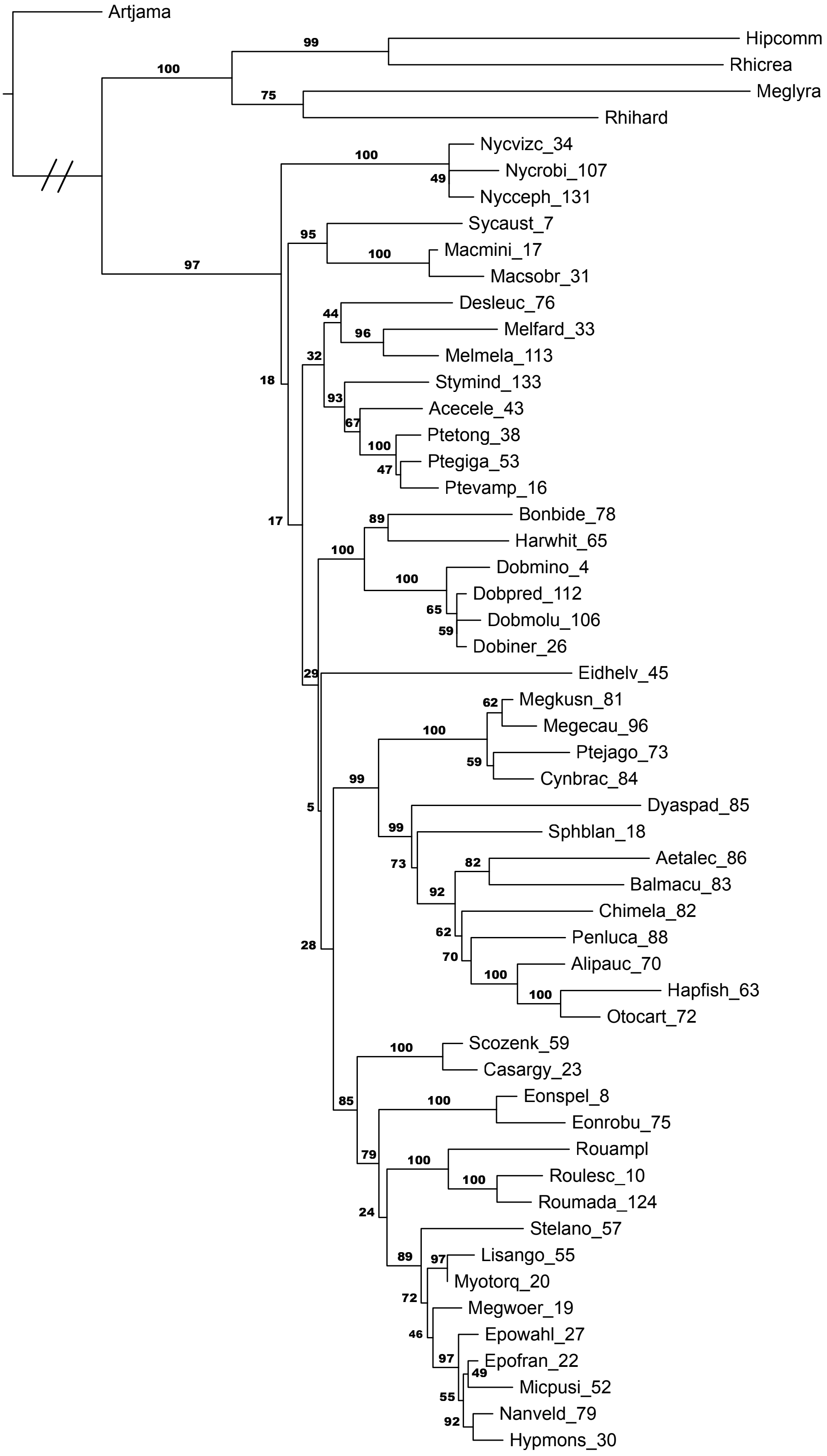

Figure S5. vWF ML gene tree with bootstrap percentages.

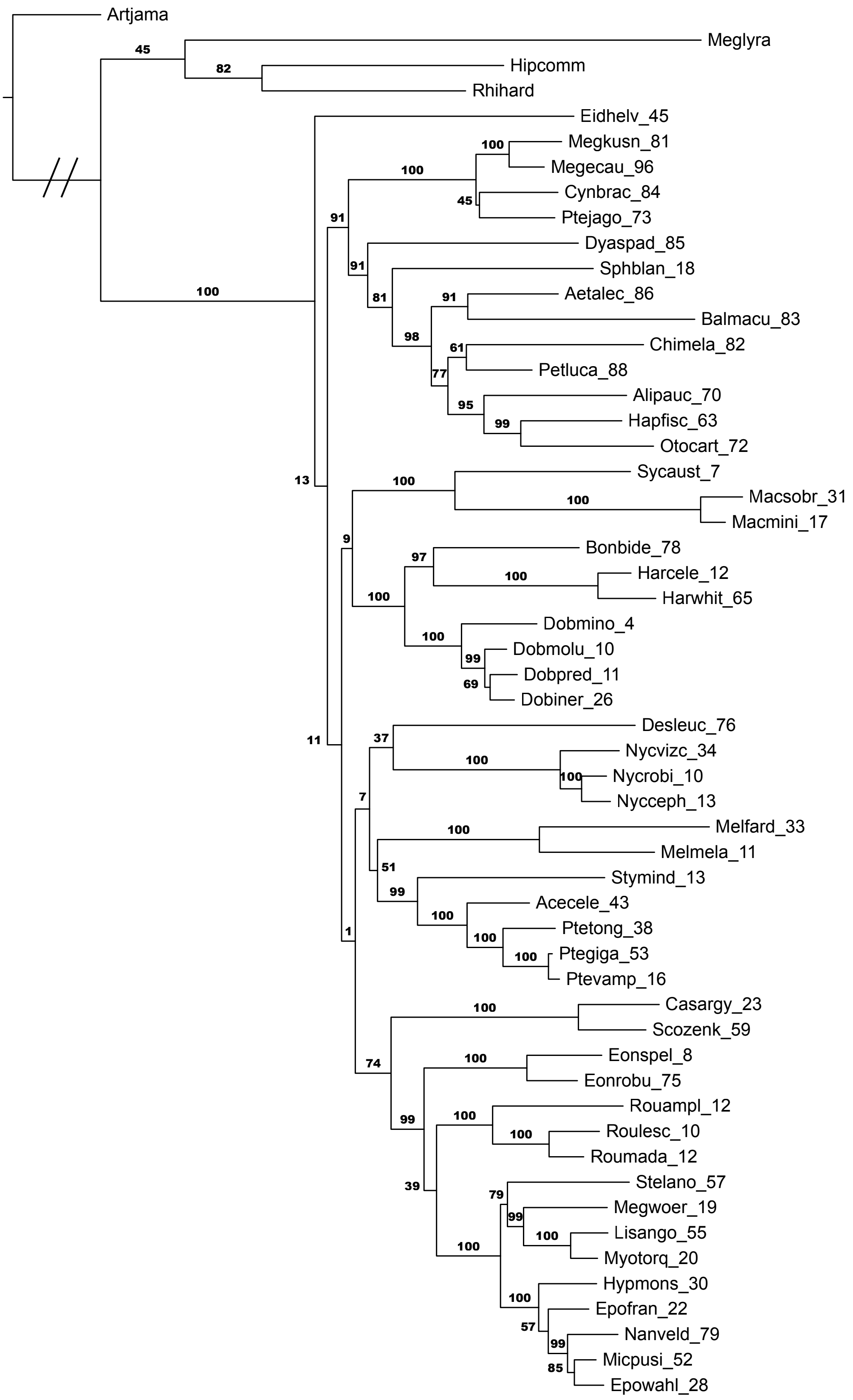

Figure S6. 12S16S ML gene tree with bootstrap percentages.

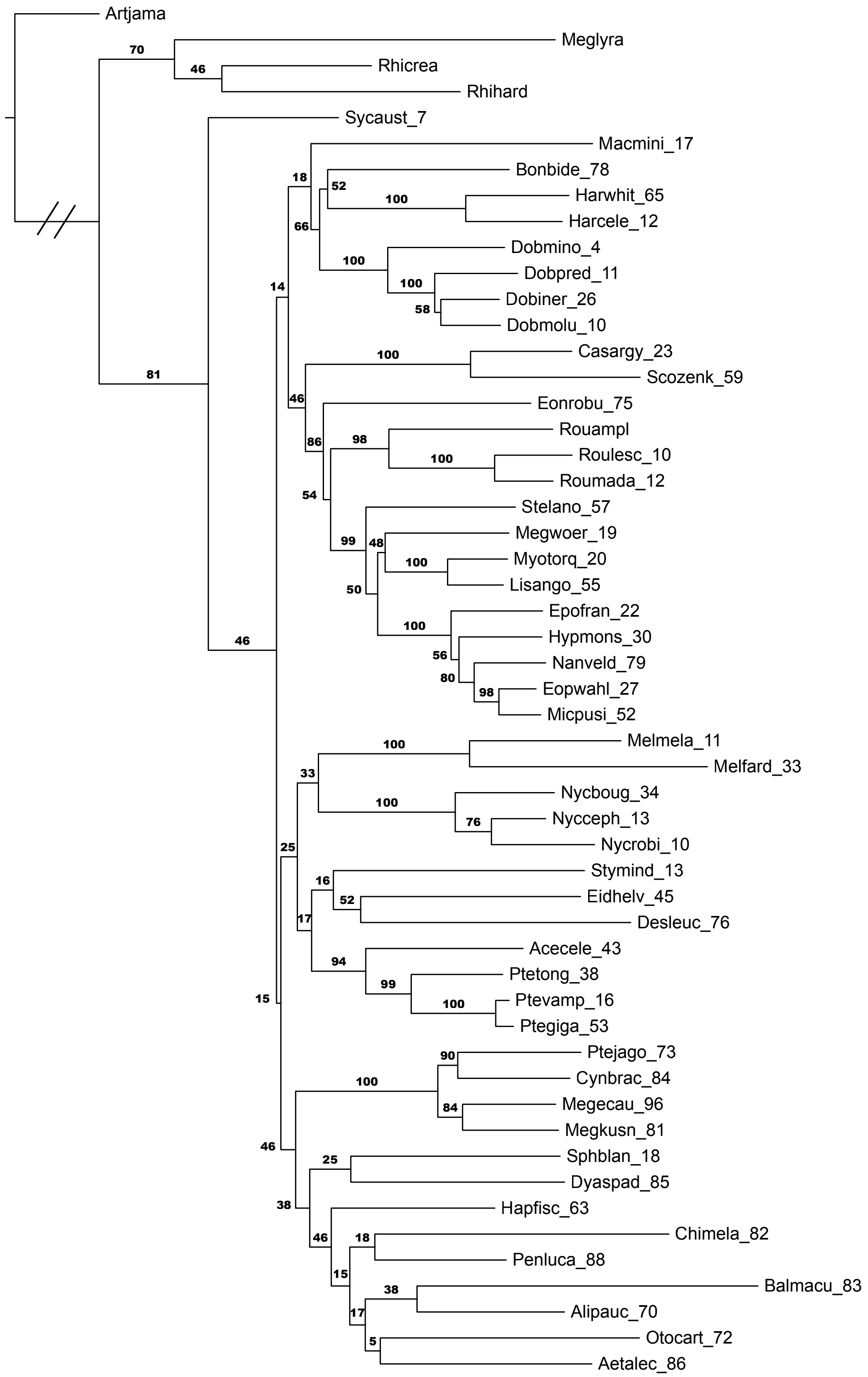

Figure S7. Cytb ML gene tree with bootstrap percentages.
