# Supplementary material for: Evolutionary relationships of the old world fruit bats (Chiroptera, Pteropodidae): Another star phylogeny?
Source: BMC Evol Biol. 2011 Sep 30;11:281. doi: 10.1186/1471-2148-11-281 (PMC3199269; doi:10.1186/1471-2148-11-281)
Supplement: Additional file 4 — Trees obtained with the exclusion of 3rd codon positions of Cytbrd. Figure S8 illustrates the MP tree with Bremer decay values and Figure S9 illustrates the ML bootstrap tree. [file 1471-2148-11-281-S4.PDF]

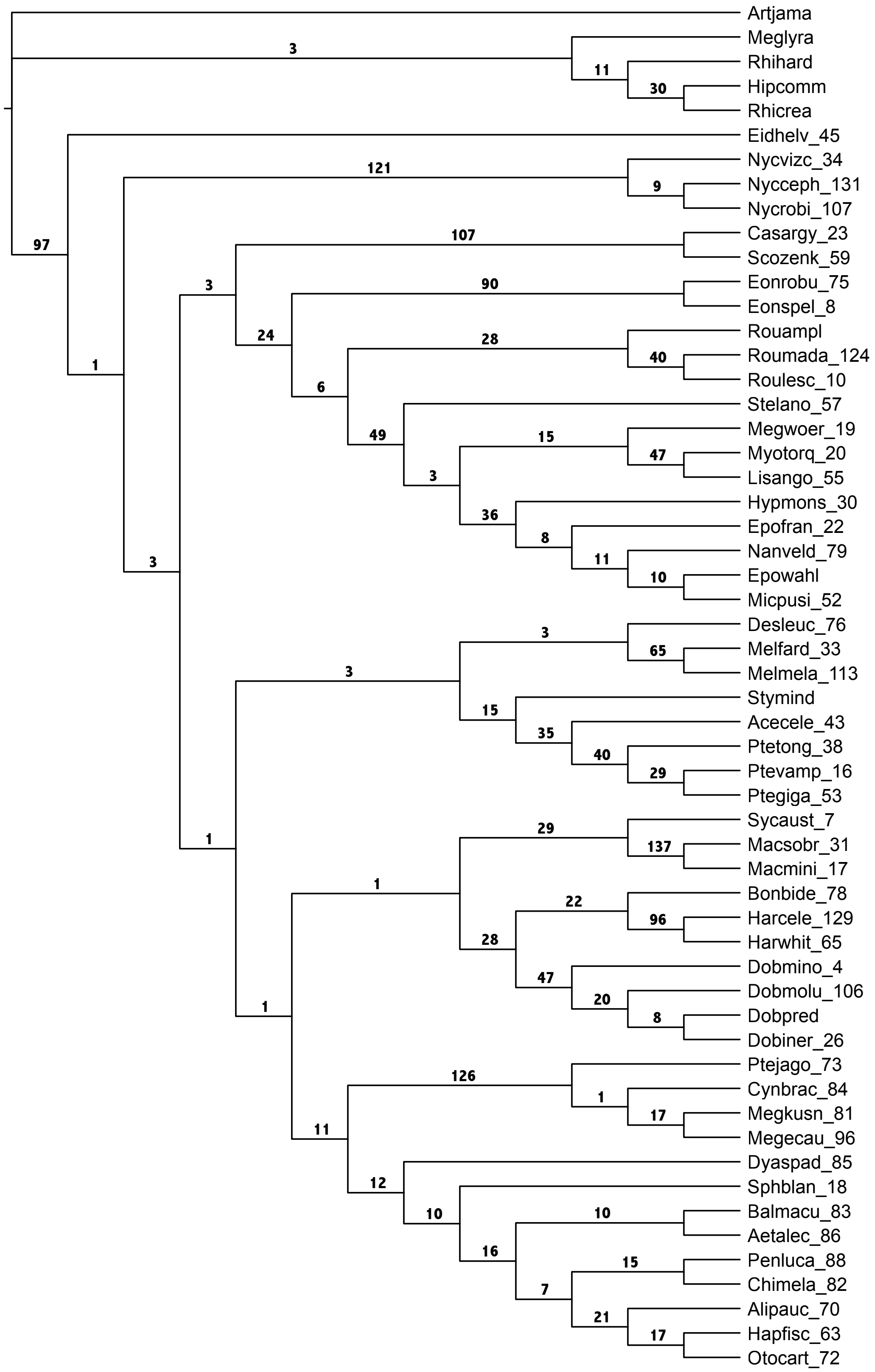

Figure S8. MP tree obtained with the combined dataset 1 when Cytb 3rd codon positions were eliminated from the analysis. Numbers above nodes represent Bremer decay values.

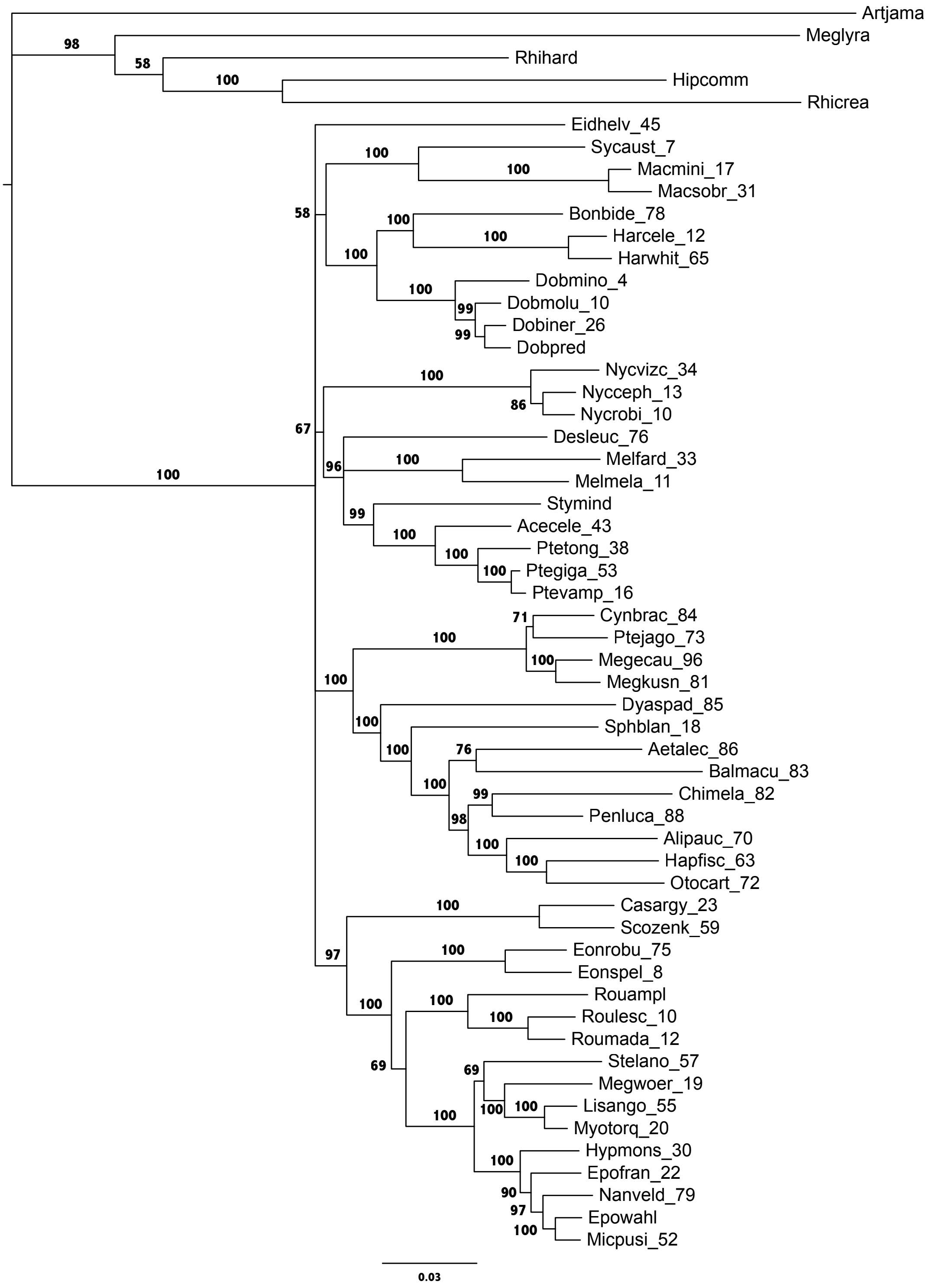

Figure S10. ML bootstrap tree obtained with the combined dataset, using optimal partition scheme (scheme 6) and substitution models, with the Cytb 3rd codon position partition eliminated.
