# Supplementary material for: Evolutionary relationships of the old world fruit bats (Chiroptera, Pteropodidae): Another star phylogeny?
Source: BMC Evol Biol. 2011 Sep 30;11:281. doi: 10.1186/1471-2148-11-281 (PMC3199269; doi:10.1186/1471-2148-11-281)
Supplement: Additional file 5 — ML topologies obtained with different data partition schemes. Figure S9 illustrating resumed ML trees obtained with dataset 1 under alternative partition schemes as described in Table 4 (main text). [file 1471-2148-11-281-S5.PDF]

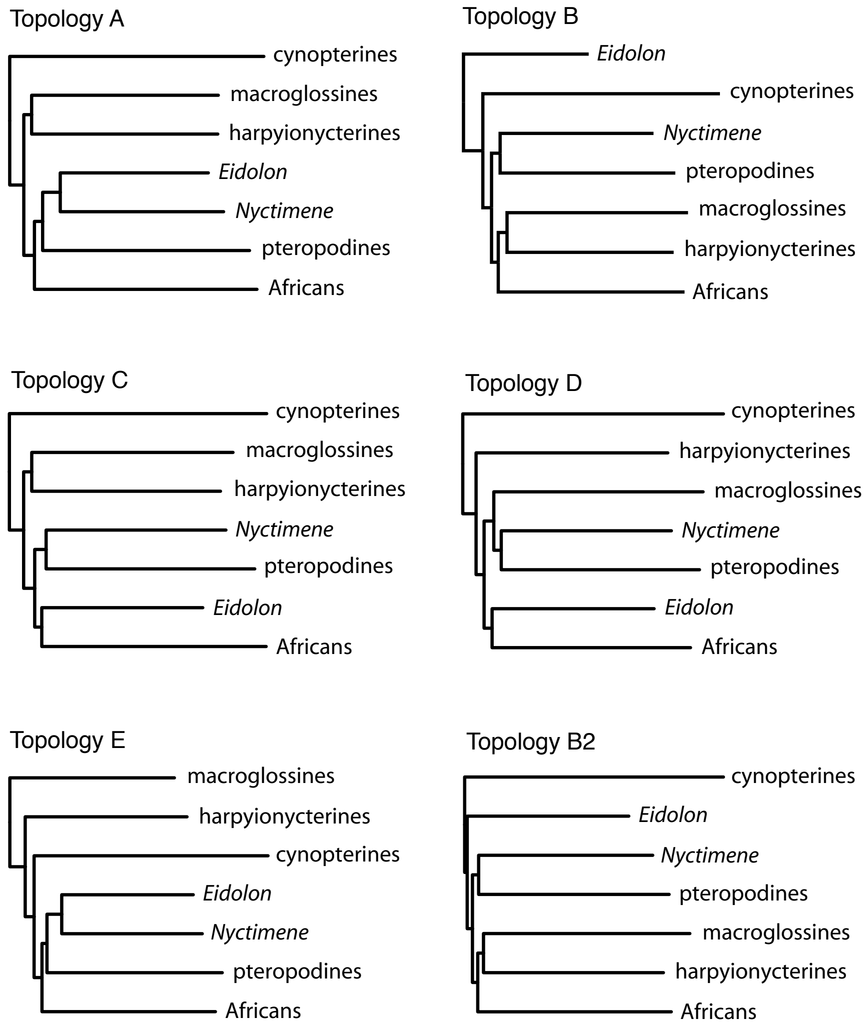

Figure S9. Topologies obtained by ML using alternative partition schemes. For details on partition scheme used to obtain each of the topologies refer to text and table 4. Topology B2 represents the one obtained using optimal partition scheme and substitution models.
