# Supplementary material for: Evolutionary relationships of the old world fruit bats (Chiroptera, Pteropodidae): Another star phylogeny?
Source: BMC Evol Biol. 2011 Sep 30;11:281. doi: 10.1186/1471-2148-11-281 (PMC3199269; doi:10.1186/1471-2148-11-281)
Supplement: Additional file 6 — Substitution models and parameters. Table S2 listing optimal substitution models and parameters for each partition under scheme 6. [file 1471-2148-11-281-S6.PDF]

Table S2. Optimal substitution models and parameters for each partition in scheme 6

partition	model	CG	alpha	rate
RAG1_12	TVM+G	0.484	0.1	0.025
RAG1_3	J3+G	0.611	0.924	0.272
RAG2_12	HKY+G	0.45	0.102	0.045
RAG2_3	HKY+G	0.453	1.348	0.203
vWF_12	J1+G	0.515	0.31	0.082
vWF_3	J2+G	0.848	0.656	0.425
BRCA1_12	J2+G	0.42	1.446	0.115
BRCA1_3	HKY+G	0.274	2.753	0.134
12S16S	GTR+G	0.405	0.201	0.767
Cytb_12	J2+G	0.442	0.105	0.301
Cytb_3	J1+G	0.428	0.678	14.123
