# Supplementary material for: Evolutionary relationships of the old world fruit bats (Chiroptera, Pteropodidae): Another star phylogeny?
Source: BMC Evol Biol. 2011 Sep 30;11:281. doi: 10.1186/1471-2148-11-281 (PMC3199269; doi:10.1186/1471-2148-11-281)
Supplement: Additional file 7 — P values of tests for zero-length branches. Table S3 showing P values of the SH and the AU tests for zero-length branches based on the combined dataset 1 for each main pteropodid node labeled on Figure 2 (main text). [file 1471-2148-11-281-S7.PDF]

Table S3. P values of the SH and the AU tests for zero-length branches based on the combined dataset 1

node[a]	pSH	pAU
A	0.673	0.21
B	0.0769	0.0175
C	0.1029	0.0389
D	0.1105	0.039
E	0.1137	0.063

[a] Nodes are labeled in Figure 2 of the manuscript.
