# Supplementary material for: Evolutionary relationships of the old world fruit bats (Chiroptera, Pteropodidae): Another star phylogeny?
Source: BMC Evol Biol. 2011 Sep 30;11:281. doi: 10.1186/1471-2148-11-281 (PMC3199269; doi:10.1186/1471-2148-11-281)
Supplement: Additional file 8 — Sequences used in this study. List of samples with Genbank accession numbers of sequences used in this study (Table S4). [file 1471-2148-11-281-S8.PDF]

Table S4. Sequences used in this study.

Species	Voucher ID	RAG1	RAG2	vWF	cytb	12S	16S	BRCA1
Acerodon celebensis	AMNH 272877	EU617946	EU617896	EU617928	GQ410231	**JN398167**	**JN398167**	**JN398239**
Aethalops alecto	ROM 102164	GQ410263	GQ410240	GQ410286	GQ410218	GQ410312	GQ410335	**JN398260**
Alionycteris paucidentata	FMNH 148099	GQ410267	GQ410244	GQ410290	GQ410222	GQ410316	GQ410339	**JN398248**
Aproteles bulmerae	GenBank	-	-	-	-	U93066	AF293645	-
Balionycteris maculata	ROM 102014	GQ410272	GQ410249	GQ410295	GQ410227	GQ410321	GQ410337	**JN398257**
Boneia bidens	Unc. (ZMA23100)	FJ218468	FJ218464	FJ218471	FJ218481	FJ218475	FJ218475	-
Casinycteris argynnis	AMNH 269915	**JN398284**	**JN398301**	**JN398268**	**JN398197**	**JN398168**	**JN398168**	**JN398264**
Chironax melanocephalus	ROM 101945	GQ410265	GQ410242	GQ410288	GQ410220	GQ410314	GQ410335	**JN398256**
Cynopterus brachyotis	ROM 102015	GQ410256	GQ410233	GQ410279	GQ410210	GQ410303	GQ410327	**JN398258**
Desmalopex leucopterus	FMNH EAR1697	UE617966	UE617915	UE617929	**JN398198**	**JN398169**	**JN398169**	**JN398252**
Dobsonia inermis	AMNH 275730	EU617948	EU617898	DQ445686	DQ445704	FJ218476	FJ218476	**JN398234**
Dobsonia minor	MVZ 140208	FJ218467	FJ218463	DQ445701	DQ445705	FJ218477	FJ218477	-
Dobsonia moluccensis	AM M20735	EU617949	EU617899	EU617930	FJ218484	FJ218472	**JN398196**	**JN398220**
Dobsonia praedatrix	USNM 580023	**JN398285**	**JN398302**	**JN398269**	**JN398199**	**JN398170**	**JN398170**	**JN398222**[a]
Dyacopterus spadiceus	ROM 102017	GQ410275	GQ410252	GQ410298	GQ410230	GQ410324	GQ410347*	**JN398259**
Eidolon helvum	CM 102021	UE617950	UE617900	UE617931	**JN398200**	**JN398171**	**JN398171**	**JN398240**
Eonycteris robusta	FMNH 178362	**JN398286**	**JN398303**	**JN398270**	**JN398201**	**JN398172**	**JN398172**	**JN398251**
Eonycteris spelaea	MVZ 176487	EU617951	EU617901	DQ445684	FJ218482	**JN398173**	**JN398173**	**JN398254**
Epomophorus wahlbergi	FMNH 177209	UE617953	EU617903	DQ445691	DQ445706	**JN398174**	**JN398174**	**JN398266**[b]
Epomops franqueti	AMNH 269902	**JN398287**	**JN398304**	**JN398271**	**JN398202**	**JN398175**	**JN398175**	**JN398233**
Haplonycteris fischeri	FMNH146627	GQ410270	GQ410247	GQ410293	GQ410225	GQ410319	GQ410342	**JN398245**
Harpyionycteris celebensis	Unc. (129)	**JN398288**	-	-	**JN398203**	FJ218473	FJ218473	**JN398225**[c]
Harpyionycteris whiteheadi	FMNH 146646	EU617954	EU617904	DQ445690	DQ445708	FJ218474	FJ218474	**JN398246**
Hypsignathus monstrosus	AMCC 116499	**JN398289**	**JN398305**	**JN398272**	**JN398204**	**JN398176**	**JN398176**	**JN398235**
Latidens salimalii	Unc. (126433)	-	-	-	GQ410217	GQ410311	-	-
Lissonycteris angolensis	CM 102135	**JN398290**	**JN398306**	**JN398273**	**JN398205**	**JN398177**	**JN398177**	**JN398243**
Macroglossus minimus	AMNH 275761	EU617955	EU617905	DQ445693	**JN398206**	**JN398178**	**JN398178**	**JN398229**
Macroglossus sobrinus	AMNH 272191	**JN398291**	**JN398307**	**JN398274**	-	**JN398179**	**JN398179**	**JN398236**
Megaerops ecaudatus	ROM 113028	GQ410260	GQ410237	GQ410283	GQ410214	GQ410308	GQ410332	**JN398262**
Megaerops kusnotoi	ROM 101944	GQ410261	GQ410238	GQ410284	GQ410215	GQ410309	GQ410333	**JN398255**
Megaloglossus woermanni	AMNH 268358	EU617956	EU617906	DQ445702	DQ445710	**JN398180**	**JN398180**	**JN398231**
Melonycteris fardoulisi	AMNH 275744	EU617957	EU617907	DQ445699	FJ218478	**JN398181**	**JN398181**	**JN398237**
Melonycteris melanops	USNM 580029	FJ218465	FJ218461	FJ218469	**JN398207**	**JN398182**	**JN398182**	**JN398223**
Micropteropus pusillus	CM 113563	**JN398292**	**JN398308**	**JN398275**	**JN398208**	**JN398183**	**JN398183**	**JN398241**
Myonycteris torquata	AMNH 268362	EU617958	EU617908	DQ445700	FJ218483	**JN398184**	**JN398184**	**JN398232**
Notopteris macdonaldi	Genbank	-	-	-	-	U93057	AF293642	-
Nanonycteris veldkampii	ROM 100560	**JN398293**	**JN398309**	**JN398276**	**JN398209**	**JN398185**	**JN398185**	**JN398253**
Nyctimene cephalotes	Unc. (131)	**JN398294**	-	**JN398277***	**JN398210**	**JN398186**	**JN398186**	**JN398226**
Nyctimene robinsoni	AM M22990	GQ410276	GQ410253	GQ410299	AF144066*	GQ410325	GQ410348	**JN398221**
Nyctimene vizcaccia	AMCC 124208	EU617959	EU617904	DQ445698	DQ445711	**JN398187**	**JN398187**	**JN398238**
Otopteropus cartilagonodus	FMNH 175391	GQ410269	GQ410246	GQ410292	GQ410224	GQ410318	GQ410341	**JN398249**
Penthetor lucasi	ROM 102183	GQ410262	GQ410239	GQ410285	GQ410216	GQ410310	GQ410334	**JN398261**
Pteralopex atrata	GenBank	-	-	-	-	U93069	AF293643	-
Ptenochirus jagori	FMNH 175395	EU617960	EU617910	DQ445696	FJ218480	GQ410304	GQ410328	**JN398250**
Pteropus giganteus	CM 92205	EU617964	EU617913	EU617935	**JN398211**	**JN398314**	AY011170[d]	**JN398242**
Pteropus tonganus	AMNH 272873	EU617976	EU617924	DQ445695	**JN398213**	**JN398188**	**JN398188**	**JN398267***
Pteropus vampyrus	AMNH 272871	**JN398295**	**JN398310**	**JN398278**	**JN398212**	**JN398189**	**JN398189**	**JN398228**
Rousettus amplexicaudatus	GenBank	AF447512	AF447529	AY057836	AB046329	U93070	AF203742	AF447500
Rousettus leschenaultii	MVZ 176490	**JN398300**	**JN398313**	**JN398283**	**JN398218**	**JN398190**		**JN398219**
Rousettus madagascariensis	USNM 449206	**JN398296**	**JN398311**	**JN398279**	**JN398214**	**JN398191**	**JN398191**	**JN398224**
Scotonycteris zenkeri	CM 107997	**JN398297**	**JN398312**	**JN398280**	**JN398216**	**JN398192**	**JN398192**	**JN398265**
Sphaerias blanfordi	AMNH274187	GQ410274	GQ410251	GQ410297	GQ410229	GQ410323	GQ410346	**JN398230**
Stenonycteris lanosus	CM 102148	**JN398298**	**JN400925**	**JN398281**	**JN398215**	**JN398193**	**JN398193**	**JN398244**
Styloctenium mindorensis	Unc. (133)	**JN398299**	-	**JN398282**	**JN398217**	**JN398194**	**JN398194**	**JN398227***
Syconycteris australis	MVZ 140265	FJ218466	FJ218462	FJ218470	FJ218479	**JN398195**	**JN398195**	**JN398247**
Thoopterus nigrescens	GenBank	-	-	-	-	U93067	AF293646	-
Rhinopoma hardwickei	GenBank	AF447518	AF447535	AF447551	AY629005	AF263232	AF263232	AF447504
Megaderma lyra	GenBank	AF203757	AF203767	U31616	DQ888678	AF069538	AF069538	AF203749
Hipposideros commersoni	GenBank	AF203760	AF203770	AF203778	-	AY395856	AY395856	AF203752
Artibeus jamaicensis	GenBank	AY834655	AY834663	AY834737	AF061340	AF061340	AF061340	AY834647
Rhinolophus creaghi	GenBank	AF447511	AF447528	AF447546	EF108163	-	-	AF447499

Sequences marked with asterisks are shorter than the standard fragment size obtained for the locus. Some sequences were obtained from a different voucher specimen: [a]USMN 580022, [b]FMNH JCK4822, [c]Unc. (130), and [d]GenBank. Voucher ID letters refer to: American Museum of Natural History (AMNH), Field Museum of Natural History (FMNH), Royal Ontario Museum (ROM), Museum of Vertebrate Museum (MVZ), Australian Museum (AM), National Museum of Natural History (USMN), Carnegie Museum of Natural History (CM).
