# Supplementary material for: Evolutionary relationships of the old world fruit bats (Chiroptera, Pteropodidae): Another star phylogeny?
Source: BMC Evol Biol. 2011 Sep 30;11:281. doi: 10.1186/1471-2148-11-281 (PMC3199269; doi:10.1186/1471-2148-11-281)
Supplement: Additional file 9 — Areas and generic distributions used in the biogeography analysis. Table S5 lists area units and Table S6 the generic distribution in those areas as used in the biogeography analysis. [file 1471-2148-11-281-S9.PDF]

Table S5. Area units used in the biogeographic analysis

Code	Description
A	African continent
B	Madagascar and surrounding Islands of the Indic Ocean
C	West and South Asia, from the Arabic Peninsula to India and Sri Lanka
D	Himalayan and Indochinese regions according to Corbet and Hill (1992)
E	Sundaic Region (Corbet and Hill, 1992)
F	Philippines, except Palawan
G	Wallacea, including Lesser Sunda Islands, Moluccas and Sulawesi
H	New Guinea and Melanesia Islands
I	Australia
J	Micronesia
K	Polynesia

Table S6. Geographic distribution of pteropodid genera.

	Africa	Madagascar + islands	West South Asia	Himalayan + Indochina	Sundaic	Philippine	Wallacean	New Guinea	Australia	Micronesia	Polynesia
Acerodon						F	G				
Aethalops					E		G				
Alionycteris						F					
Aproteles								H			
Balionycteris					E						
Boneia							G				
Casinycteris	A										
Chironax					E		G				
Cynopterus			C	D	E	F	G				
Desmalopex						F					
Dobsonia							G	H	I		
Dyacopterus					E	F					
Eidolon	A	B									
Eonycteris			C	D	E	F	G				
Epomophorus	A										
Epomops	A										
Haplonycteris						F					
Harpyionycteris						F	G				
Hypsignathus	A										
Latidens			C								
Lissonycteris	A										
Macroglossus				D	E	F					
Megaerops				D	E		G				
Megaloglossus	A										
Melonycteris								H			

Micropteropus	A										
Myonycteris	A										
Nanonycteris	A										
Notopteris								H			K
Nyctimene						F	G	H	I		
Otopteropus						F					
Penthetor					E						
Ptenochirus						F					
Pteralopex								H		J	
Pteropus		B	C	D	E	F	G	H	I	J	K
Rousettus amplexicaudatus				D	E	F	G	H			
Rousettus leschenaultii	A	B	C	D	E	F	G	H			
Rousettus madagascariensis	A	B	C	D	E	F	G	H			
Scotonycteris	A										
Sphaerias				D							
Stenonycteris	A										
Styloctenium						F	G				
Syconycteris								H	I		
Thoopterus							G				
